# Supplementary figures and images for: Clinical characteristics and factors associated with COVID-19-related mortality and hospital admission during the first two epidemic waves in 5 rural provinces in Indonesia: A retrospective cohort study
Source: PLoS One. 2023 Mar 30;18(3):e0283805. doi: 10.1371/journal.pone.0283805 (PMC10062642; doi:10.1371/journal.pone.0283805)

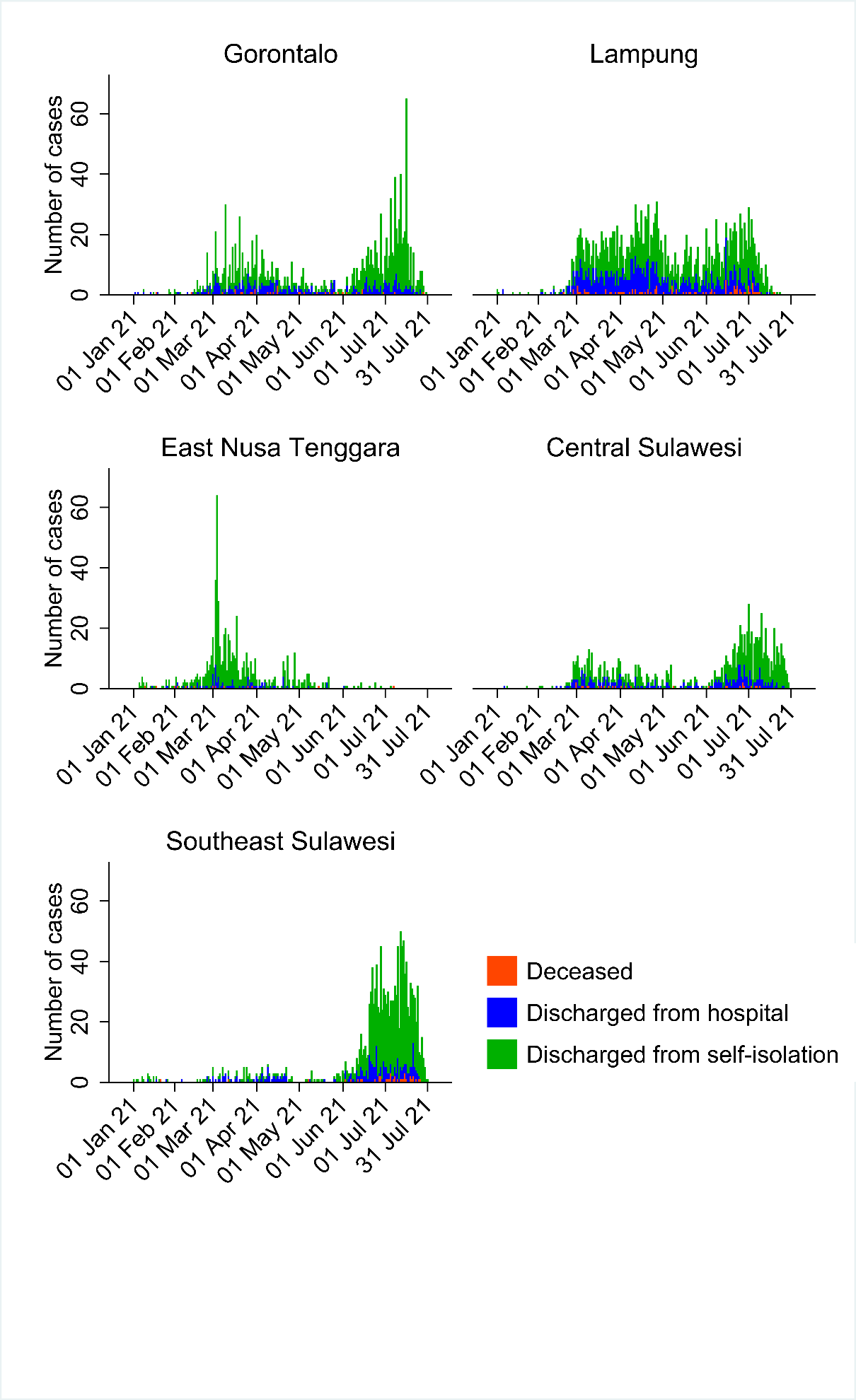

Supplement: S1 Fig — (TIF) [file pone.0283805.s001.tif]
