# Supplementary material for: Clinical characteristics and factors associated with COVID-19-related mortality and hospital admission during the first two epidemic waves in 5 rural provinces in Indonesia: A retrospective cohort study
Source: PLoS One. 2023 Mar 30;18(3):e0283805. doi: 10.1371/journal.pone.0283805 (PMC10062642; doi:10.1371/journal.pone.0283805)
Supplement: S1 Table — (DOCX) [file pone.0283805.s002.docx]

**S1 Table. Population number and number of health care workers by province**

| **Province** | **Population number** | **Number of doctors per 100,000 population** | **Number of nurses per 100,000 population** | **Number of midwives per 100,000 population** | **Number of public health officer per 100,000 population** |
| --- | --- | --- | --- | --- | --- |
| Lampung | 9,007,848 | 4.97 | 10.52 | 19.18 | 9.20 |
| Gorontalo | 1,171,681 | 6.57 | 17.41 | 9.13 | 51.40 |
| Central Sulawesi | 2,985,734 | 8.47 | 27.46 | 13.73 | 47.60 |
| Southeast Sulawesi | 2,624,875 | 12.59 | 36.51 | 13.03 | 52.50 |
| East Nusa Tenggara | 5,325,566 | 6.80 | 25.63 | 12.74 | 14.00 |
| Jakarta | 10,562,088 | 44.81 | 48.30 | 30.70 | NA |

NA=Not available
