# Supplementary material for: Clinical characteristics and factors associated with COVID-19-related mortality and hospital admission during the first two epidemic waves in 5 rural provinces in Indonesia: A retrospective cohort study
Source: PLoS One. 2023 Mar 30;18(3):e0283805. doi: 10.1371/journal.pone.0283805 (PMC10062642; doi:10.1371/journal.pone.0283805)
Supplement: S2 Table — (DOCX) [file pone.0283805.s003.docx]

**S2 Table. Number of cases, population number and COVID-19 incidence rate by province**

| **Province** | **Number of cases** | **Population number** | **Incidence rate per 100,000 population** |
| --- | --- | --- | --- |
| Lampung | 2,146 | 9,007,848 | 23.8 |
| Gorontalo | 1,356 | 1,171,681 | 115.7 |
| Central Sulawesi | 987 | 2,985,734 | 33.1 |
| Southeast Sulawesi | 1,436 | 2,624,875 | 54.7 |
| East Nusa Tenggara | 658 | 5,325,566 | 12.4 |
