# Supplementary material for: Clinical characteristics and factors associated with COVID-19-related mortality and hospital admission during the first two epidemic waves in 5 rural provinces in Indonesia: A retrospective cohort study
Source: PLoS One. 2023 Mar 30;18(3):e0283805. doi: 10.1371/journal.pone.0283805 (PMC10062642; doi:10.1371/journal.pone.0283805)
Supplement: S3 Table — (DOCX) [file pone.0283805.s004.docx]

**S3 Table. Bivariable mixed effects logistic regression analysis showing factors associated with risk of COVID-19 mortality and hospitalisation in five rural provinces, Indonesia**

|  | **Mortality** | | **Hospitalisation** | |
| --- | --- | --- | --- | --- |
|  | **OR (95% CI)** | **p value** | **OR (95% CI)** | **p value** |
| Age group, years |  |  |  |  |
| 0-19 | 1 (reference) |  | 1 (reference) |  |
| 20-29 | 2.20 (0.61-7.89) | 0.228 | 1.13 (0.88-1.44) | 0.340 |
| 30-39 | 2.40 (0.67-8.51) | 0.177 | **1.31 (1.03-1.67)** | **0.029** |
| 40-49 | **5.87 (1.76-19.61)** | **0.004** | **2.02 (1.59-2.57)** | **<0.001** |
| 50-59 | **15.44 (4.81-49.51)** | **<0.001** | **4.31 (3.41-5.45)** | **<0.001** |
| 60-69 | **32.85 (10.24-105.34)** | **<0.001** | **6.25 (4.83-8.10)** | **<0.001** |
| ≥70 | **51.65 (15.74-169.51)** | **<0.001** | **7.52 (5.41-10.47)** | **<0.001** |
| Sex |  |  |  |  |
| Female | 1 (reference) |  | 1 (reference) |  |
| Male | **1.38 (1.05-1.83)** | **0.023** | 0.97 (0.87-1.09) | 0.623 |
| Clinical diagnosis with pneumonia |  |  |  |  |
| No | 1 (reference) |  | 1 (reference) |  |
| Yes | **16.13 (11.51-22.62)** | **<0.001** | **22.50 (16.01-31.62)** | **<0.001** |
| Hypertension |  |  |  |  |
| No | 1 (reference) |  | 1 (reference) |  |
| Yes | **6.14 (4.52-8.34)** | **<0.001** | **5.42 (4.49-6.54)** | **<0.001** |
| Diabetes |  |  |  |  |
| No | 1 (reference) |  | 1 (reference) |  |
| Yes | **7.80 (5.63-10.81)** | **<0.001** | **6.44 (5.11-8.11)** | **<0.001** |
| Cardiac diseases |  |  |  |  |
| No | 1 (reference) |  | 1 (reference) |  |
| Yes | **7.16 (4.72-10.85)** | **<0.001** | **5.96 (4.35-8.15)** | **<0.001** |
| COPD |  |  |  |  |
| No | 1 (reference) |  | 1 (reference) |  |
| Yes | **2.19 (1.49-2.88)** | **<0.001** | **15.04 (6.67-33.90)** | **<0.001** |
| Chronic kidney diseases |  |  |  |  |
| No | 1 (reference) |  | 1 (reference) |  |
| Yes | **8.90 (4.45-17.83)** | **<0.001** | **9.34 (4.57-19.11)** | **<0.001** |
| Liver diseases |  |  |  |  |
| No | 1 (reference) |  | 1 (reference) |  |
| Yes | **12.91 (3.98-41.90)** | **<0.001** | **11.40 (3.10-41.89)** | **<0.001** |
| Malignancy |  |  |  |  |
| No | 1 (reference) |  | 1 (reference) |  |
| Yes | **7.66 (2.52-23.29)** | **<0.001** | **3.13 (1.27-7.69)** | **0.013** |
| Immunocompromised |  |  |  |  |
| No | 1 (reference) |  | 1 (reference) |  |
| Yes | NA | NA | **5.26 (1.92-14.42)** | **<0.001** |
| Number of comorbidities |  |  |  |  |
| 0 | 1 (reference) |  | 1 (reference) |  |
| 1 | **7.48 (5.37-10.41)** | **<0.001** | **6.29 (5.32-7.44)** | **<0.001** |
| >1 | **19.74 (13.55-28.75)** | **<0.001** | **10.25 (7.62-13.78)** | **<0.001** |
| Density of doctors | 0.63 (0.28-1.39) | 0.250 | 0.47 (0.13-1.71) | 0.254 |
| Density of nurses | **0.82 (0.68-0.99)** | **0.037** | **0.72 (0.53-0.97)** | **0.029** |
| Density of midwives | **2.00 (1.38-2.91)** | **<0.001** | 2.07 (0.78-5.48) | 0.144 |
| Density of public health officers | 0.91 (0.83-1.01) | 0.068 | 0.98 (0.80-1.19) | 0.822 |

Province was treated as the random effect variable.

OR: odds ratio.

NA: Not Applicable due to 0 value of observation with immunocompromised condition among deceased cases.

Health care workers density was calculated as ratio of each type of health workers per 100,000 population
