# Supplementary material for: Clinical characteristics and factors associated with COVID-19-related mortality and hospital admission during the first two epidemic waves in 5 rural provinces in Indonesia: A retrospective cohort study
Source: PLoS One. 2023 Mar 30;18(3):e0283805. doi: 10.1371/journal.pone.0283805 (PMC10062642; doi:10.1371/journal.pone.0283805)
Supplement: S4 Table — (DOCX) [file pone.0283805.s005.docx]

**S4 Table. Mixed effects logistic regression multivariable models assessing association between and mortality and hospitalisation with number of comorbidities in five rural provinces, Indonesia**

|  | **Mortality** | | **Hospitalisation** | |
| --- | --- | --- | --- | --- |
|  | **aOR (95% CI)** | **p value** | **aOR (95% CI)** | **p value** |
| Number of comorbidities |  |  |  |  |
| 0 | 1 (reference) |  | 1 (reference) |  |
| 1 | **2.56 (1.68-3.90)** | **<0.0001** | **3·56 (2·91-4·35)** | **<0.001** |
| >1 | **4.95 (3.01-8.15)** | **<0.0001** | **4·46 (3·14-6·35)** | **<0.001** |

Model was adjusted for age, sex, pneumonia, and number of health care workers per 100,000 population. Province was treated as the random effect variable. aOR: adjusted odds ratio.
