# Supplementary material for: Clinical characteristics and factors associated with COVID-19-related mortality and hospital admission during the first two epidemic waves in 5 rural provinces in Indonesia: A retrospective cohort study
Source: PLoS One. 2023 Mar 30;18(3):e0283805. doi: 10.1371/journal.pone.0283805 (PMC10062642; doi:10.1371/journal.pone.0283805)
Supplement: S1 Checklist — (DOCX) [file pone.0283805.s006.docx]

STROBE Statement—checklist of items that should be included in reports of observational studies

|  | Item No. | Recommendation | Page  No. | Relevant text from manuscript |
| --- | --- | --- | --- | --- |
| **Title and abstract** | 1 | (*a*) Indicate the study’s design with a commonly used term in the title or the abstract | Title, page 1 | “a retrospective cohort study” |
|  |  | (*b*) Provide in the abstract an informative and balanced summary of what was done and what was found | Abstract, page 2 | “This retrospective cohort included individuals diagnosed with COVID-19 based on polymerase chain reaction or rapid antigen diagnostic test, from five rural provinces in Indonesia. We extracted demographic and clinical data, including hospitalisation and mortality from a new piloted COVID-19 information system named Sistem Informasi Surveilans Epidemiologi (SISUGI). We used mixed-effect logistic regression to examine factors associated with COVID-19-related mortality and hospitalisation.  The risk of COVID-19-related mortality and hospitalisation was associated with higher age, pre-existing chronic comorbidities, and clinical pneumonia. The findings highlight the need for prioritising enhanced context-specific public health action to reduce mortality and hospitalisation risk among older and comorbid rural populations.” |
| Introduction | | | |  |
| Background/rationale | 2 | Explain the scientific background and rationale for the investigation being reported | Background, paragraphs 1-4, page 3-4 |  |
| Objectives | 3 | State specific objectives, including any prespecified hypotheses | Background, paragraph 4, page 4 | “This study utilised the epidemiological surveillance data recorded in the SISUGI, to better understand clinical characteristics and factors associated with COVID-19-related mortality and hospitalisation in five rural provinces in Indonesia, from 1 January to 31 July 2021.” |
| Methods | | | |  |
| Study design | 4 | Present key elements of study design early in the paper | Methods, study design and participants, page 4 | “This was a retrospective cohort analysis to assess demographic and clinical factors associated with mortality and hospitalisation of adults and children diagnosed with COVID-19 in five provinces in Indonesia”. |
| Setting | 5 | Describe the setting, locations, and relevant dates, including periods of recruitment, exposure, follow-up, and data collection | Methods, study design and participants and Data collection, page 4-5 |  |
| Participants | 6 | (*a*) *Cohort study*—Give the eligibility criteria, and the sources and methods of selection of participants. Describe methods of follow-up  *Case-control study*—Give the eligibility criteria, and the sources and methods of case ascertainment and control selection. Give the rationale for the choice of cases and controls  *Cross-sectional study*—Give the eligibility criteria, and the sources and methods of selection of participants | Methods, study design and participants, page 5  N/a | “The study population included individuals diagnosed with COVID-19 based on either a PCR test or Ag RDT done in either health facilities or laboratory from Lampung, Gorontalo, Central Sulawesi, Southeast Sulawesi, and East Nusa Tenggara povinces between January to July 2021.” |
|  |  | (*b*) *Cohort study*—For matched studies, give matching criteria and number of exposed and unexposed  *Case-control study*—For matched studies, give matching criteria and the number of controls per case | N/a |  |
| Variables | 7 | Clearly define all outcomes, exposures, predictors, potential confounders, and effect modifiers. Give diagnostic criteria, if applicable | Methods, data collection, page 5 | “Data regarding dates of test, onset of illness, SARS-CoV-2 Ag RDT and PCR testing, and outcomes (mortality and hospital admission) were recorded, along with age, gender, symptoms, and comorbidities. Comorbidities were recorded based on clinical assessments at admission or patient reporting. Fever was defined as axillary temperature of ≥ 38°C. Clinical diagnosis of pneumonia (indicative of moderate or severe disease [28]) was determined by the treating physician, based on clinical and radiological evaluation [29]. Individual-level epidemiological data of all COVID-19 confirmed cases between January to July 2021 were then extracted from the SISUGI database. Province-level data were collected from various government sources. Data on population numbers by province in 2020 were collected from the Statistics Bureau Database [30]. Data on the number of doctors, nurses, midwives per province, as per October 2021 were collected from the Indonesia Ministry of Health records [31].” |
| Data sources/ measurement | 8* | For each variable of interest, give sources of data and details of methods of assessment (measurement). Describe comparability of assessment methods if there is more than one group | Methods, data collection, page 5 | “Each health facility had a surveillance officer responsible for conducting an epidemiological investigation of each confirmed case via interview using a standard form. Completed forms were submitted to the SISUGI database. Data regarding dates of test, onset of illness, SARS-CoV-2 Ag RDT and PCR testing, and outcomes (mortality and hospital admission) were recorded, along with age, gender, symptoms, and comorbidities. Comorbidities were recorded based on clinical assessments at admission or patient reporting. Fever was defined as axillary temperature of ≥ 38°C. Clinical diagnosis of pneumonia (indicative of moderate or severe disease [28]) was determined by the treating physician, based on clinical and radiological evaluation [29]. Individual-level epidemiological data of all COVID-19 confirmed cases between January to July 2021 were then extracted from the SISUGI database. Province-level data were collected from various government sources. Data on population numbers by province in 2020 were collected from the Statistics Bureau Database [30]. Data on the number of doctors, nurses, midwives per province, as per October 2021 were collected from the Indonesia Ministry of Health records [31].” |
| Bias | 9 | Describe any efforts to address potential sources of bias | Methods, statistical analysis, page 5-6 | “Due to the high proportion of missing data needed to define the time from onset to admission, this variable was not analysed.  We used bivariable and multivariable mixed effects logistic regression models to determine the risk of death and hospitalisation, expressed as odds ratios with 95% confidence intervals. Province was treated as the random effect variable to adjust for clustering of observations within provinces. We did null model analysis (no predictor was added) and the result justified the use of the mixed effects models. All independent variables with p-value <0.10 in bivariable analysis were included in the multivariable models. Sex was treated as a “forced variable” (a strong potential confounder to be included in multivariable models, regardless of its p-value in bivariable analysis). Final model selection was informed by likelihood ratio tests. We reported two final multivariable logistic regression models. In the first model, we assessed the effect of each type of comorbidity (hypertension, diabetes, cardiac disease, chronic obstructive pulmonary disease (COPD), chronic kidney disease, immunocompromised status, and liver disease), along with other demographic and clinical factors. In the second model, we assessed the effect of the number of comorbidities (0, 1 or >1), controlling for other demographic and clinical factors. We used interaction terms to examine potential effect modification by age and sex. We set statistical significance at 0.05, and all tests were two sided. All analyses were done in Stata/IC 15.1 (StataCorp, College Station, TX, USA). This study is reported as per Strengthening the Reporting of Observational Studies in Epidemiology (STROBE) guidelines [31].” |
| Study size | 10 | Explain how the study size was arrived at | Methods, study design and participants and data collection, page 5 | “The study population included individuals diagnosed with COVID-19 based on either a PCR test or Ag RDT done in either health facilities or laboratory from Lampung, Gorontalo, Central Sulawesi, Southeast Sulawesi, and East Nusa Tenggara povinces between January to July 2021.  Individual-level epidemiological data of all COVID-19 confirmed cases between January to July 2021 were then extracted from the SISUGI database. Province-level data were collected from various government sources.” |

Continued on next page

| Quantitative variables | 11 | Explain how quantitative variables were handled in the analyses. If applicable, describe which groupings were chosen and why | Methods, statistical analysis, page 5-6 | “Descriptive statistics included proportions for categorical variables and medians and interquartile ranges (IQRs) for continuous variables. We used the Mann-Whitney U test to compare median different of age between deceased and recovered cases, and between hospitalised and non-hospitalised cases. For categorical variables, we used Chi-squared test, or Fisher’s exact test to compare other characteristics between deceased and recovered cases, and between hospitalised and non-hospitalised cases. Due to the high proportion of missing data needed to define the time from onset to admission, this variable was not analysed. |
| --- | --- | --- | --- | --- |
| Statistical methods | 12 | (*a*) Describe all statistical methods, including those used to control for confounding | Methods, statistical analysis, page 6 | “Descriptive statistics included proportions for categorical variables and medians and interquartile ranges (IQRs) for continuous variables. We used the Mann-Whitney U test, Chi-squared test, or Fisher’s exact test to compare characteristics between deceased and recovered cases, and between hospitalised and non-hospitalised cases. Due to the high proportion of missing data needed to define the time from onset to admission, this variable was not analysed.  We used bivariable and multivariable mixed effects logistic regression models to determine the risk of death and hospitalisation, expressed as odds ratios with 95% confidence intervals. Province was treated as the random effect variable to adjust for clustering of observations within provinces. We did null model analysis (no predictor was added) and the result justified the use of the mixed effects models. All independent variables with p-value <0.10 in bivariable analysis were included in the multivariable models. Sex was treated as a “forced variable” (a strong potential confounder to be included in multivariable models, regardless of its p-value in bivariable analysis). Final model selection was informed by likelihood ratio tests. We reported two final multivariable logistic regression models. In the first model, we assessed the effect of each type of comorbidity (hypertension, diabetes, cardiac disease, chronic obstructive pulmonary disease (COPD), chronic kidney disease, immunocompromised status, and liver disease), along with other demographic and clinical factors. In the second model, we assessed the effect of the number of comorbidities (0, 1 or >1), controlling for other demographic and clinical factors. We used interaction terms to examine potential effect modification by age and sex. We set statistical significance at 0.05, and all tests were two sided. All analyses were done in Stata/IC 15.1 (StataCorp, College Station, TX, USA). This study is reported as per Strengthening the Reporting of Observational Studies in Epidemiology (STROBE) guidelines [31].”” |
|  |  | (*b*) Describe any methods used to examine subgroups and interactions | Methods, statistical analysis, page 6 | “We reported two final multivariable logistic regression models. In the first model, we assessed the effect of each type of comorbidity (hypertension, diabetes, cardiac disease, chronic obstructive pulmonary disease (COPD), chronic kidney disease, immunocompromised status, and liver disease), along with other demographic and clinical factors. In the second model, we assessed the effect of the number of comorbidities (0, 1 or >1), controlling for other demographic and clinical factors. We used interaction terms to examine potential effect modification by age and sex.” |
|  |  | (*c*) Explain how missing data were addressed | N/a | “The completeness of data was high, with less than 1% missing data across the variables analysed in the study” |
|  |  | (*d*) *Cohort study*—If applicable, explain how loss to follow-up was addressed  *Case-control study*—If applicable, explain how matching of cases and controls was addressed  *Cross-sectional study*—If applicable, describe analytical methods taking account of sampling strategy | N/a | - |
|  |  | (*e*) Describe any sensitivity analyses | N/a | - |
| Results | | | | |
| Participants | 13* | (a) Report numbers of individuals at each stage of study—eg numbers potentially eligible, examined for eligibility, confirmed eligible, included in the study, completing follow-up, and analysed | Results, paragraph 1-2, page 6 | “Between 1 January and 31 July 2021, a total of 6,583 confirmed COVID-19 cases were recorded on the SISUGI. The completeness of data was high, with less than 1% missing data across the variables analysed in the study. The study flow chart and completeness of data are presented in Fig 2.  Table 1 presents the demographic and clinical characteristics of the 6,583 cases included in the analysis. The highest proportion of cases were reported in Lampung (32.6%, 2,146/6,583), and the lowest was in East Nusa Tenggara (10.0%, 658/6,583). The median age was 37 years (IQR 26-51, range 0.1-96), with 825 (12.6%) under 20 years, and 3,371 (51.2%) were females. The incidence rate ranged from 12 per 100,000 populations in Lampung to 116 per 100,000 populations in Gorontalo (S2 Table).” |
|  |  | (b) Give reasons for non-participation at each stage | N/a |  |
|  |  | (c) Consider use of a flow diagram | N/a |  |
| Descriptive data | 14* | (a) Give characteristics of study participants (eg demographic, clinical, social) and information on exposures and potential confounders | Results, paragraphs 2-5 (page 6-7), Table 1, Fig 3, and S1 Fig |  |
|  |  | (b) Indicate number of participants with missing data for each variable of interest | Fig 2 |  |
|  |  | (c) *Cohort study*—Summarise follow-up time (eg, average and total amount) | N/a |  |
| Outcome data | 15* | *Cohort study*—Report numbers of outcome events or summary measures over time | Results, Paragraph 3 (Page 7) and S1 Fig | “Of 6,583 cases included in the analysis, 205 (3.1%) were deceased, and 1,727 (26.2%) were hospitalised. The number of COVID-19-related deaths and hospital admissions varied over time and by province (S1 Fig).” |
|  |  | *Case-control study—*Report numbers in each exposure category, or summary measures of exposure | N/a |  |
|  |  | *Cross-sectional study—*Report numbers of outcome events or summary measures | N/a |  |
| Main results | 16 | (*a*) Give unadjusted estimates and, if applicable, confounder-adjusted estimates and their precision (eg, 95% confidence interval). Make clear which confounders were adjusted for and why they were included | Results paragraph 7-10 (page 10-12), Table 3, S3-4 Table | “In bivariable mixed effects logistic regression analysis (S3 Table), the risk of death was associated with older age, male sex, clinical diagnosis with pneumonia, number of pre-existing comorbidities, hypertension, diabetes, cardiac disease, COPD, chronic kidney disease, liver diseases, malignancy, lower number of nurses per 100,000 population, and higher number of midwives per 100,000 population. The risk of being hospitalised was associated with older age, clinical diagnosis with pneumonia, number of pre-existing comorbidities, hypertension, diabetes, cardiac disease, COPD, chronic kidney disease, liver diseases, malignancy, immunocompromised, and lower number of nurses per 100,000 populations.  Based on the first multivariable mixed effects logistic regression model (Table 3), the risk of death increased for age groups 50-59 years (aOR 6.5, 95%CI 2.0-21.3), 60-69 years (aOR 10.8, 95%CI 3.2-35.8), and ≥70 years (aOR 18.3, 95%CI 5.3-63.2), compared to <20 years; for cases with pre-existing diabetes (aOR 2.0, 95%CI 1.3-3.2), chronic kidney disease (aOR 6.1, 95%CI 2.5-14.6), liver diseases (aOR 10.3, 95% CI 1.8-58.7), or malignancy (aOR 7.4, 95% CI 1.5-36.9); for patients who had pneumonia (aOR 8.0, 95%CI 5.3-12.0), compared to those without pre-existing comorbidities.  The risk of being hospitalised increased for age groups 40-49 years (aOR 1.7, 95%CI 1.3-2.2), 50-59 years (aOR 2.9, 95%CI 2.2-3.8), 60-69 years (aOR 3.3, 95%CI 2.4-4.4), and ≥70 years (aOR 3.7, 95%CI 2.5-5.5), compared to <20 years; for cases with pre-existing hypertension (aOR 2.4, 95%CI 1.9-3.0), diabetes (aOR 2.2, 95%CI 1.7-2.9), cardiac disease (aOR 2.1, 95% CI 1.4-3.1), COPD (aOR 7.5, 95% CI 2.3-24.1), chronic kidney disease (aOR 3.6, 95%CI 1.6-8.2), liver diseases (aOR 7.5, 95% CI 1.8-31.0), malignancy (aOR 3.4, 95% CI 1.1-10.4), or immunocompromised (aOR 6.9, 95% CI 2.4-20.2); for patients who had pneumonia (aOR 15.3, 95%CI 10.7-21.8).  In the second multivariable model (S4 Table), we found that both the risk of death and hospitalisation increased with the number of pre-existing comorbidities, after controlling for age, sex, pneumonia, and number of health care workers. Sex and number of health care workers at province-level were not associated with COVID-19-related hospitalisation and mortality. Sex and age were not found to be an effect modifier in either model.” |
|  |  | (*b*) Report category boundaries when continuous variables were categorized | N/a |  |
|  |  | (*c*) If relevant, consider translating estimates of relative risk into absolute risk for a meaningful time period | N/a |  |

Continued on next page

| Other analyses | 17 | Report other analyses done—eg analyses of subgroups and interactions, and sensitivity analyses | N/a |  |
| --- | --- | --- | --- | --- |
| Discussion | | | | |
| Key results | 18 | Summarise key results with reference to study objectives | Discussion, paragraph 1, page 12-13 | “This retrospective cohort study described the clinical characteristics and factors associated with risk of COVID-19-related mortality and hospitalisation based on epidemiological surveillance data of confirmed COVID-19 cases reported from five rural provinces of Indonesia, during January to July 2021. This analysis was one of the first COVID-19 epidemiological studies reporting data from rural settings of Indonesia. The observed disease pattern broadly reflects that reported globally, with patients usually presenting multiple symptoms of fever, cough, malaise and/or shortness of breath. The overall mortality and hospitalisation rate was 3.1% (205/6,583) and 26.2% (1,727/6,583), respectively. The age-specific mortality and hospitalisation rate for children under five years old was 0.9% (2/215) and 17.2% (37/215), respectively. The mortality and hospitalisation rate were highly heterogeneous, with the highest rate was reported in Lampung (4.3% mortality and 36.1% hospitalisation), and the lowest rate in East Nusa Tenggara (1.9% mortality and 13.4% hospitalisation). Higher risk of mortality and hospital admission was associated with increased age, clinical diagnosis with pneumonia, presence of pre-existing diabetes, chronic kidney disease, liver disease, and malignancy. Presence of pre-existing hypertension, cardiac diseases, COPD, and immunocompromised conditions were associated with higher risk of hospitalisation, but not associated with mortality. Gender and number of health care workers at province-level were not associated with COVID-19-related mortality and hospitalisation.” |
| Limitations | 19 | Discuss limitations of the study, taking into account sources of potential bias or imprecision. Discuss both direction and magnitude of any potential bias | Discussion, paragraph 6, page 14-15 | “This study has limitations. The retrospective design and reliance on routine surveillance data meant that data were incomplete or unavailable for some key baseline variables (e.g., date of symptom onset, date of hospital admission and outcome, vital signs, and disease severity classification). The imperfect contact tracing, testing, and reporting activities could result in underreporting of cases, especially for asymptomatic and mild cases which thus results in overestimation of rates of mortality and hospitalisation rate. Comorbidities were often self-reported or could be under-diagnosed, potentially resulting in underreporting and hence underestimation of effect sizes. Findings from this study may not reflect the mortality and hospitalisation rate, and risk factors associated with COVID-19-related outcomes in other LMIC settings with different demographics, non-infectious diseases prevalence, and different health care capacity.” |
| Interpretation | 20 | Give a cautious overall interpretation of results considering objectives, limitations, multiplicity of analyses, results from similar studies, and other relevant evidence | Discussion paragraphs 2-6, page 13-14 |  |
| Generalisability | 21 | Discuss the generalisability (external validity) of the study results | Conclusion, page 15 |  |
| Other information | |  | | |
| Funding | 22 | Give the source of funding and the role of the funders for the present study and, if applicable, for the original study on which the present article is based | Funding, page 16 | “This study received no specific grant from any funding agency in the public, commercial or not-for-profit sectors. The funders had no role in study design, data collection and analysis, decision to publish, or preparation of the manuscript.” |

*Give information separately for cases and controls in case-control studies and, if applicable, for exposed and unexposed groups in cohort and cross-sectional studies.

**Note:** An Explanation and Elaboration article discusses each checklist item and gives methodological background and published examples of transparent reporting. The STROBE checklist is best used in conjunction with this article (freely available on the Web sites of PLoS Medicine at http://www.plosmedicine.org/, Annals of Internal Medicine at http://www.annals.org/, and Epidemiology at http://www.epidem.com/). Information on the STROBE Initiative is available at www.strobe-statement.org.
