# Supplementary material for: Clinical characteristics and factors associated with COVID-19-related mortality and hospital admission during the first two epidemic waves in 5 rural provinces in Indonesia: A retrospective cohort study
Source: PLoS One. 2023 Mar 30;18(3):e0283805. doi: 10.1371/journal.pone.0283805 (PMC10062642; doi:10.1371/journal.pone.0283805)
Supplement: Attachment — Submitted filename: PONE-D-22-31666_reviewer Review.pdf [file pone.0283805.s007.pdf]

PLOS ONE

Clinical characteristics and factors associated with COVID-19-related mortality and hospital admission in 5 rural provinces in Indonesia: a retrospective cohort study

--Manuscript Draft--

**Manuscript Number:**	PONE-D-22-31666
**Article Type:**	Research Article
**Full Title:**	Clinical characteristics and factors associated with COVID-19-related mortality and hospital admission in 5 rural provinces in Indonesia: a retrospective cohort study
**Short Title:**	Clinical characteristics and factors associated with COVID-19-related mortality and hospital admission
**Corresponding Author:**	Henry Surendra, PhD<br>Oxford University Clinical Research Unit<br>Jakarta, Jakarta INDONESIA
**Keywords:**	COVID-19; coronavirus; mortality; hospitalisation; epidemiology; Indonesia
**Abstract:**	Background<br><br>Data on coronavirus disease 2019 (COVID-19) clinical characteristics and severity from resource-limited settings are limited. This study examined clinical characteristics and factors associated with COVID-19 mortality and hospitalisation in rural settings of Indonesia, from 1 January to 31 July, 2021.<br><br>Methods<br><br>This retrospective cohort included individuals diagnosed with COVID-19 based on polymerase chain reaction or rapid antigen diagnostic test, from Lampung, Gorontalo, Central Sulawesi, Southeast Sulawesi, and East Nusa Tenggara Provinces, Indonesia. We extracted demographic and clinical data, including hospitalisation and mortality from COVID-19 surveillance records. We used mixed-effect logistic regression to examine factors associated with COVID-19-related mortality and hospitalisation.<br><br>Results<br><br>Of 6,583 confirmed cases, 205 (3%) died and 1,727 (26%) were hospitalised. The median age was 37 years (Interquartile range 26-51), with 825 (13%) under 20 years, and 3,371 (51%) females. Most cases were symptomatic (4,533; 69%); 319 (5%) had a clinical diagnosis of pneumonia and 945 (14%) presented with at least one pre-existing comorbidity. Age-specific mortality rates were 1% (2/340) for 0-4 years; 0% (0/112) for 5-9 years; 0% (1/498) for 10-19 years; 1% (11/1,385) for 20-29 years; 1% (12/1,382) for 30-39 years; 2% (23/1,095) for 40-49 years; 5% (57/1,064) for 50-59 years; 11% (62/576) for 60-69 years; 16% (37/232) for ≥70 years. Older age, pre-existing diabetes, liver diseases, malignancy, and pneumonia were associated with higher risk of mortality and hospitalisation. Pre-existing hypertension, cardiac diseases, chronic kidney disease, COPD, and immunocompromised condition were associated with risk of hospitalisation but not with mortality.<br><br>Conclusion<br><br>Clinical characteristics and risk factors of severe COVID-19 outcomes in rural provinces were broadly similar to those in urban settings. The risk of COVID-19-related mortality and hospitalisation was associated with higher age, pre-existing chronic comorbidities, and clinical presentation of pneumonia.
**Order of Authors:**	Henry Surendra, PhD
	C Yekti Praptiningsih, MPH
	Arina M Ersanti
	Mariati Rahmat, MPH

Powered by Editorial Manager® and ProduXion Manager® from Aries Systems Corporation

	Widia Noviyanti
	Joshua AD Harmani, MD
	Erni NA Mansur
	Yana Y Suleman
	Sitti Sudrani
	Rosalina Rosalina
	Ismen Mukhtar, MSc
	Dian Rosadi, MPH
	Lukman Fauzi, MPH
	Iqbal RF Elyazar, PhD
	William A Hawley, PhD
	Hariadi Wibisono, PhD
**Additional Information:**	
**Question**	**Response**
**Financial Disclosure**<br><br>Enter a financial disclosure statement that describes the sources of funding for the work included in this submission. Review the submission guidelines for detailed requirements. View published research articles from *PLOS ONE* for specific examples.<br><br>This statement is required for submission and **will appear in the published article** if the submission is accepted. Please make sure it is accurate.	This study received no specific grant from any funding agency in the public, commercial or not-for-profit sectors. HS and IRFE were funded by the Wellcome (UK) Africa Asia Programme Vietnam (106680/Z/14/Z). The funders had no role in study design, data collection and analysis, decision to publish, or preparation of the manuscript.

**Unfunded studies**<br>Enter: *The author(s) received no specific funding for this work.*<br><br>**Funded studies**<br>Enter a statement with the following details:<br>• Initials of the authors who received each award<br>• Grant numbers awarded to each author<br>• The full name of each funder<br>• URL of each funder website<br>• Did the sponsors or funders play any role in the study design, data collection and analysis, decision to publish, or preparation of the manuscript?<br>• **NO** - Include this sentence at the end of your statement: *The funders had no role in study design, data collection and analysis, decision to publish, or preparation of the manuscript.*<br>• **YES** - Specify the role(s) played.<br><br>* typeset	
**Competing Interests**<br><br>Use the instructions below to enter a competing interest statement for this submission. On behalf of all authors, disclose any competing interests that could be perceived to bias this work—acknowledging all financial support and any other relevant financial or non-financial competing interests.<br><br>This statement is required for submission and **will appear in the published article** if the submission is accepted. Please make sure it is accurate and that any funding sources listed in your Funding Information later in the submission form are also declared in your Financial Disclosure statement.<br><br>View published research articles from *PLOS ONE* for specific examples.	The authors have declared that no competing interests exist

**NO authors have competing interests**<br><br>Enter: *The authors have declared that no competing interests exist.*<br><br>**Authors with competing interests**<br><br>Enter competing interest details beginning with this statement:<br><br>*I have read the journal's policy and the authors of this manuscript have the following competing interests: [insert competing interests here]*<br><br>* typeset	
**Ethics Statement**<br><br>Enter an ethics statement for this submission. This statement is required if the study involved:<br><br>• Human participants<br>• Human specimens or tissue<br>• Vertebrate animals or cephalopods<br>• Vertebrate embryos or tissues<br>• Field research<br><br>Write "N/A" if the submission does not require an ethics statement.<br><br>General guidance is provided below. Consult the submission guidelines for detailed instructions. **Make sure that all information entered here is included in the Methods section of the manuscript.**	This study was approved by the Health Research Ethics Committee of Universitas Negeri Semarang (300/2/KEPK/EC/2022). The requirement for patient consent was waived as this was a secondary analysis of anonymised routine surveillance data.

Powered by Editorial Manager® and ProduXion Manager® from Aries Systems Corporation

**Format for specific study types**<br><br>**Human Subject Research (involving human participants and/or tissue)**<br>• Give the name of the institutional review board or ethics committee that approved the study<br>• Include the approval number and/or a statement indicating approval of this research<br>• Indicate the form of consent obtained (written/oral) or the reason that consent was not obtained (e.g. the data were analyzed anonymously)<br><br>**Animal Research (involving vertebrate animals, embryos or tissues)**<br>• Provide the name of the Institutional Animal Care and Use Committee (IACUC) or other relevant ethics board that reviewed the study protocol, and indicate whether they approved this research or granted a formal waiver of ethical approval<br>• Include an approval number if one was obtained<br>• If the study involved *non-human primates*, add *additional details* about animal welfare and steps taken to ameliorate suffering<br>• If anesthesia, euthanasia, or any kind of animal sacrifice is part of the study, include briefly which substances and/or methods were applied<br><br>**Field Research**<br><br>Include the following details if this study involves the collection of plant, animal, or other materials from a natural setting:<br>• Field permit number<br>• Name of the institution or relevant body that granted permission	
**Data Availability**<br><br>Authors are required to make all data underlying the findings described fully available, without restriction, and from the time of publication. PLOS allows rare exceptions to address legal and ethical concerns. See the PLOS Data Policy and FAQ for detailed information.	No - some restrictions will apply

A Data Availability Statement describing where the data can be found is required at submission. Your answers to this question constitute the Data Availability Statement and **will be published in the article**, if accepted.<br><br>**Important:** Stating 'data available on request from the author' is not sufficient. If your data are only available upon request, select 'No' for the first question and explain your exceptional situation in the text box.<br><br>Do the authors confirm that all data underlying the findings described in their manuscript are fully available without restriction?	
**Describe where the data may be found in full sentences. If you are copying our sample text, replace any instances of XXX with the appropriate details.**<br><br>• If the data are **held or will be held in a public repository**, include URLs, accession numbers or DOIs. If this information will only be available after acceptance, indicate this by ticking the box below. For example: *All XXX files are available from the XXX database (accession number(s) XXX, XXX.).*<br>• If the data are all contained **within the manuscript and/or Supporting Information files**, enter the following: *All relevant data are within the manuscript and its Supporting Information files.*<br>• If neither of these applies but you are able to provide **details of access elsewhere**, with or without limitations, please do so. For example:<br><br>*Data cannot be shared publicly because of [XXX]. Data are available from the XXX Institutional Data Access / Ethics Committee (contact via XXX) for researchers who meet the criteria for access to confidential data.*<br><br>*The data underlying the results presented in the study are available from (include the name of the third party*	After publication, the datasets used for this study will be made available to others on reasonable requests to the corresponding author, including a detailed research proposal, study objectives and statistical analysis plan.

and contact information or URL).<br>• This text is appropriate if the data are owned by a third party and authors do not have permission to share the data.<br><br>* typeset	
Additional data availability information:	

Manuscript

Click here to access/download;Manuscript;Surendra et al. Manuscript with tables.docx

Clinical characteristics and factors associated with COVID-19-related mortality and hospital admission in 5 rural provinces in Indonesia: a retrospective cohort study

Authors: Henry Surendra[1,2*], C Yekti Praptiningsih[3,4], Arina M Ersanti[3], Mariati Rahmat[3,5], Widia Noviyanti[3], Joshua AD Harmani[3], Erni NA Mansur[6], Yana Y Suleman[6], Sitti Sudrani[7], Rosalina Rosalina[8], Ismen Mukhtar[9], Dian Rosadi[10], Lukman Fauzi[11], Iqbal RF Elyazar[1], William A Hawley[4], Hariadi Wibisono[3]

[1] Oxford University Clinical Research Unit Indonesia, Jakarta, Indonesia
[2] Monash University Indonesia, Tangerang Selatan, Indonesia
[3] Perhimpunan Ahli Epidemiologi Indonesia, Jakarta, Indonesia
[4] US Centers for Disease Control and Prevention, Jakarta, Indonesia
[5] Dinas Kesehatan Kabupaten Sinjai, Sinjai, Indonesia
[6] Dinas Kesehatan Provinsi Gorontalo, Gorontalo, Indonesia
[7] Dinas Kesehatan Provinsi Sulawesi Tengah, Manado, Indonesia
[8] Dinas Kesehatan Provinsi Sulawesi Tenggara, Kendari, Indonesia
[9] Dinas Kesehatan Provinsi Lampung, Bandar Lampung, Indonesia
[10] Faculty of Medicine, Universitas Lambung Mangkurat, Banjarmasin, Indonesia
[11] Public Health Science Department, Universitas Negeri Semarang, Semarang, Indonesia

Corresponding author:
Henry Surendra PhD.
Oxford University Clinical Research Unit Indonesia
Jalan Diponegoro 69
10430 Jakarta, Indonesia.
Phone: (021) 3910414
Email: henrysurendra.15@gmail.com

Disclaimer: The findings and conclusions in this report are those of the authors and do not necessarily represent the official position of the U.S. Centers for Disease Control and Prevention.

Abstract (282 words)

Background Data on coronavirus disease 2019 (COVID-19) clinical characteristics and severity from resource-limited settings are limited. This study examined clinical characteristics and factors associated with COVID-19 mortality and hospitalisation in rural settings of Indonesia, from 1 January to 31 July, 2021.

Methods This retrospective cohort included individuals diagnosed with COVID-19 based on polymerase chain reaction or rapid antigen diagnostic test, from Lampung, Gorontalo, Central Sulawesi, Southeast Sulawesi, and East Nusa Tenggara Provinces, Indonesia. We extracted demographic and clinical data, including hospitalisation and mortality from COVID-19 surveillance records. We used mixed-effect logistic regression to examine factors associated with COVID-19-related mortality and hospitalisation.

Results Of 6,583 confirmed cases, 205 (3%) died and 1,727 (26%) were hospitalised. The median age was 37 years (Interquartile range 26-51), with 825 (13%) under 20 years, and 3,371 (51%) females. Most cases were symptomatic (4,533; 69%); 319 (5%) had a clinical diagnosis of pneumonia and 945 (14%) presented with at least one pre-existing comorbidity. Age-specific mortality rates were 1% (2/340) for 0-4 years; 0% (0/112) for 5-9 years; 0% (1/498) for 10-19 years; 1% (11/1,385) for 20-29 years; 1% (12/1,382) for 30-39 years; 2% (23/1,095) for 40-49 years; 5% (57/1,064) for 50-59 years; 11% (62/576) for 60-69 years; 16% (37/232) for ≥70 years. Older age, pre-existing diabetes, liver diseases, malignancy, and pneumonia were associated with higher risk of mortality and hospitalisation. Pre-existing hypertension, cardiac diseases, ~~chronic kidney disease~~, COPD, and immunocompromised condition were associated with risk of hospitalisation but not with mortality.

Conclusion Clinical characteristics and risk factors of severe COVID-19 outcomes in rural provinces were broadly similar to those in urban settings. The risk of COVID-19-related mortality and hospitalisation was associated with higher age, pre-existing chronic comorbidities, and clinical presentation of pneumonia.

Keywords: COVID-19, coronavirus, mortality, hospitalisation, epidemiology, Indonesia

Background (3133 words)

The coronavirus disease 2019 (COVID-19) pandemic continues to affect the world population. Globally, there have been more than 600 million total confirmed cases, and 6.6 million deaths reported to the World Health Organization (WHO) as of 16 November 2022 [1]. As per 8 November 2022, almost 13 billion COVID-19 vaccine doses have been administered worldwide [1], yet 40,476 new COVID-19-related deaths were still being reported in the last 28 days [2].

Current evidence suggests that severe outcomes of COVID-19 have been consistently associated with older age and pre-existing chronic conditions, such as hypertension, diabetes, obesity, cardiac disease, chronic kidney disease, and liver disease [3–8]. Moreover, recent findings from US [11-12], Chile [13], Brazil [14], and including from Indonesia [15] have suggested that poor outcomes associated with COVID-19 are concentrated in groups with higher socio-demographic and health care vulnerability. However, the understanding of COVID-19 severity mostly comes from studies conducted in urban settings. Limited data exist from rural settings on COVID-19 clinical characteristics and factors associated with severity where differences in age distribution, comorbidities, access to quality health services, and other factors may influence trends regarding severe outcomes [8,15-18].

Indonesia is a low-middle income country (LMIC) that has suffered the highest number of COVID-19 confirmed cases and deaths in Southeast Asia, second only to India in Asia [2]. Since the first reported cases on 2 March 2020, Indonesia has reported a total of 6,582,291 cases and 159,253 deaths (2.4% confirmed case fatality rate) up to 16 November 2022 [19]. The majority of cases and deaths in Indonesia were reported on Java Island, a more developed setting populated by 152 million individuals (56% of Indonesia's total population). Recent studies from Indonesia's capital city of Jakarta suggested that COVID-19 disproportionately affected individuals with older age and pre-existing chronic comorbidities, as well as those areas with lower vaccine coverage and higher poverty and population density [8,15]. However, data on the impact of COVID-19 in less developed areas of Indonesia with greater geographic, cultural and socio-economic diversity, outside of the island of Java, are scarce.

The limited evidence regarding COVID-19 epidemiology and severity from low resource settings in Indonesia – especially in areas outside of Java - has been particularly associated with the lack of epidemiological data recorded by the surveillance systems in these areas. To improve quality of COVID-19 surveillance and response in Indonesia, the Indonesian

Epidemiologist Association (Perhimpunan Ahli Epidemiologi Indonesia, PAEI) in collaboration with the Ministry of Health of Indonesia implemented a new COVID-19 information system named Sistem Informasi Surveilans Epidemiologi (SISUGI) [20] in five rural provinces – East Nusa Tenggara in eastern Indonesia, Gorontalo, Central Sulawesi, and Southeast Sulawesi on the island of Sulawesi, and Lampung on the island of Sumatra (Figure 1). This study utilised the epidemiological surveillance data recorded in the SISUGI, to better understand clinical characteristics and factors associated with COVID-19-related mortality and hospitalisation in five rural provinces in Indonesia, from 1 January to 31 July 2021.

Materials and Methods

Study design and participants

This was a retrospective cohort analysis to assess demographic and clinical factors associated with mortality and hospitalisation of adults and children diagnosed with COVID-19 in five provinces in Indonesia. The study population included individuals diagnosed with COVID-19 based on either a polymerase chain reaction (PCR) test or rapid antigen diagnostic test (Ag RDT) done in either health facilities or laboratory from Lampung, Gorontalo, Central Sulawesi, Southeast Sulawesi, and East Nusa Tenggara provinces between January to July 2021. In accordance with Indonesia's national COVID-19 guidelines, individuals are categorized as COVID-19 confirmed cases if they tested positive by Ag RDT or PCR [21]. Based on data from Indonesia Central Bureau of Statistics, the majority of areas in the five provinces were categorised as rural areas i.e., Lampung (68%), Gorontalo (66%), Central Sulawesi (55%), Southeast Sulawesi (83%), and East Nusa Tenggara (92%) [22, 23]. Based on the number of doctors, nurses, midwives, and public health officer per 100,000 population (Supplementary Table 1), these five provinces were considered as rural settings with limited health care capacity. The five provinces were purposively selected as pilot project sites for the implementation SISUGI (https://sisugi.com/) developed by PAEI in collaboration with the US CDC Indonesia Country Office, South Asia Field Epidemiology and Technology Network (SAFETYNET), and Ministry of Health of the Republic of Indonesia. This study was conducted as part of a program evaluation.

Data collection

Each health facility had a surveillance officer responsible for conducting an epidemiological investigation of each confirmed case via interview using a standard form. Completed forms were submitted to the SISUGI database. Data regarding dates of test, onset of illness, SARS-CoV-2 Ag RDT and PCR testing, and outcomes (mortality and hospital admission) were recorded, along with age, gender, symptoms, and comorbidities. Comorbidities were recorded

based on clinical assessments at admission or patient reporting. Fever was defined as axillary temperature of ≥ 38°C. Clinical diagnosis of pneumonia (indicative of moderate or severe disease [24]) was determined by the treating physician, based on clinical and radiological evaluation [25]. Individual-level epidemiological data of all COVID-19 confirmed cases between January to July 2021 were then extracted from the SISUGI database. Province-level data were collected from various government sources. Data on population numbers by province in 2020 were collected from the Statistics Bureau Database [26]. Data on the number of doctors, nurses, midwives per province, as per October 2021 were collected from the Indonesia Ministry of Health records.

Statistical analysis

Descriptive statistics included proportions for categorical variables and medians and interquartile ranges (IQRs) for continuous variables. We used the Mann-Whitney U test, Chi-squared test, or Fisher's exact test to compare characteristics between deceased and recovered cases, and between hospitalised and non-hospitalised cases. Due to the high proportion of missing data needed to define the time from onset to admission, this variable was not analysed.

We used bivariable and multivariable mixed effects logistic regression models to determine the risk of death and hospitalisation, expressed as odds ratios with 95% confidence intervals. Province was treated as the random effect variable to adjust for clustering of observations within provinces. We did null model analysis (no predictor was added) and the result justified the use of the mixed effects models. All independent variables with p-value <0.10 in bivariable analysis were included in the multivariable models. Final model selection was informed by likelihood ratio tests. We reported two final multivariable logistic regression models. In the first model, we assessed the effect of each type of comorbidity (hypertension, diabetes, cardiac disease, chronic obstructive pulmonary disease (COPD), chronic kidney disease, immunocompromised status, and liver disease), and in the second model, the number of comorbidities (0, 1 or >1), along with other demographic and clinical factors. We used interaction terms to examine potential effect modification by age and sex. We set statistical significance at 0·05, and all tests were two sided. All analyses were done in Stata/IC 15.1 (StataCorp, College Station, TX, USA). This study is reported as per Strengthening the Reporting of Observational Studies in Epidemiology (STROBE) guidelines [27].

Ethics approval

This study was approved by the Health Research Ethics Committee of Universitas Negeri Semarang (300/2/KEPK/EC/2022). The requirement for patient consent was waived as this was a secondary analysis of anonymised routine surveillance data.

Consent for publication

Not applicable

Availability of data and materials

After publication, the datasets used for this study will be made available to others on reasonable requests to the corresponding author, including a detailed research proposal, study objectives and statistical analysis plan.

Conflict of interests

The authors have declared that no competing interests exist.

Funding

This study received no specific grant from any funding agency in the public, commercial or not-for-profit sectors. HS and IRFE were funded by the Wellcome (UK) Africa Asia Programme Vietnam (106680/Z/14/Z). The funders had no role in study design, data collection and analysis, decision to publish, or preparation of the manuscript.

Results

General characteristics of study participants

Between 1 January and 31 July 2021, a total of 6,583 confirmed COVID-19 cases were recorded on the SISUGI. The completeness of data was high, with less than 1% missing data across the variables analysed in the study. The study flow chart and completeness of data are presented in Figure 2.

Table 1 presents the demographic and clinical characteristics of the 6,583 cases included in the analysis. The highest proportion of cases were reported in Lampung (33%, 2,146/6,583), and the lowest was in East Nusa Tenggara (10%, 658/6,583). The median age was 37 years (IQR 26-51, range 0·1-96), with 825 (13%) under 20 years, and 3,371 (51%) were females. The incidence rate ranged from 12 per 100,000 populations in Lampung to 116 per 100,000 populations in Gorontalo (Supplementary Table 2).

Clinical characteristics and COVID-19-related mortality and hospitalisation

Of 6,583 cases included in the analysis, 205 (3%) were deceased, and 1,727 (26%) were hospitalised. The number of COVID-19-related deaths and hospital admissions varied over time and by province (Supplementary Figure 1). There were 4,533 (69%) symptomatic cases, with the most common presenting symptom being cough (47%, 3,072), followed by fever (44%, 2,894), runny nose (33%, 2,174), malaise (25%, 1,671), and others (Figure 3A). The median number of symptoms was 2 (IQR 0–4, range 1–13). There were 319 (5%) patients with a clinical diagnosis of pneumonia. Overall, 14% (945) of cases had a record of one or more pre-existing comorbidities, including hypertension (8%, 539), diabetes mellitus (6%, 365), cardiac disease (3%, 187), and others (Figure 3B).

Compared to recovered cases, deceased cases were older (median 58 vs 36 years); more likely to have more presenting symptoms (median 3 vs 2); to have pneumonia (33% vs 4%); and to have a history of any comorbidity (56% vs 12%), 1 comorbidity (34% vs 10%) or >1 comorbidities (26% vs 3%), specifically hypertension (33% vs 7%), diabetes (29% vs 5%), and cardiac disease (15% vs 3%) (Table 1).

Compared to non-hospitalised cases, hospitalised cases were older (median 50 vs 34 years); more likely to have more presenting symptoms (median 3 vs 1); to have pneumonia (16% vs 1%); and to have a history of any comorbidity (32% vs 6%), 1 comorbidity (25% vs 6%) or >1 comorbidities (10% vs 1%), specifically hypertension (19% vs 4%), diabetes (14% vs 2%), and cardiac disease (2% vs 0%) (Table 1).

Table 1. Demographics, clinical characteristics, health care workers, and COVID-19-related mortality and hospitalisation in 5 rural provinces in Indonesia, 1 January to 31 July 2021

	Total N=6,583*	Deceased			Hospitalised		
		Yes (N=205)	No (N=6,378)	p value	Yes (N=1,727)	No (N=4,856)	p value
**Demographics**							
Province				0.002			<0.0001
Lampung	2,146 (32.6)	93 (45.4%)	2,053 (32.2%)		775 (44.9%)	1,371 (28.2%)	
Gorontalo	1,356 (20.6%)	34 (16.6%)	1,322 (20.7%)		340 (19.7%)	1,016 (20.9%)	
Central Sulawesi	987 (15.0%)	27 (13.2%)	960 (15.1%)		258 (14.9%)	729 (15.0%)	
Southeast Sulawesi	1,436 (21.8%)	38 (18.5%)	1,398 (21.9%)		266 (15.4%)	1,170 (24.1%)	
East Nusa Tenggara	658 (10.0%)	13 (6.3%)	645 (10.1%)		88 (5.1%)	570 (11.7%)	
Median age (IQR), years	37 (26-51)	58 (50-68)	36 (26-51)	<0.0001	50 (33-59)	34 (25-47)	<0.0001
Age group, years				<0.0001			<0.0001
0-4	215 (3.3%)	2 (1.0%)	213 (3.3%)		37 (2.1%)	178 (3.7%)	
5-9	112 (1.7%)	0 (0.0%)	112 (1.8%)		14 (0.8%)	98 (2.0%)	
10-19	498 (7.6)	1 (0.5%)	497 (7.8%)		65 (3.8%)	433 (8.9%)	
20-29	1,385 (21.0%)	11 (5.4%)	1,374 (21.5%)		213 (12.3%)	1,172 (24.1%)	
30-39	1,382 (21.0)	12 (5.9%)	1,370 (21.5%)		249 (14.4%)	1,133 (23.3%)	
40-49	1,095 (16.6)	23 (11.2%)	1,072 (16.8%)		273 (15.8%)	822 (16.9%)	
50-59	1,064 (16.2%)	57 (27.8%)	1,007 (15.8%)		446 (25.8%)	618 (12.7%)	
60-69	576 (8.8%)	62 (30.2%)	514 (8.1%)		297 (17.2%)	279 (5.8%)	
≥70	232 (3.5%)	37 (18.1%)	195 (3.1%)		131 (7.6%)	101 (2.1%)	
Unknown	24 (0.4%)	0 (0.0%)	24 (0.4%)		2 (0.1%)	22 (0.5%)	
Gender				0.074			0.115
Female	3,371 (51.2%)	90 (43.9%)	3,281 (51.4%)		904 (52.4%)	2,467 (50.8%)	
Male	3,196 (48.6%)	115 (56.1%)	3,081 (48.3%)		822 (47.6%)	2,374 (48.9%)	
Unknown	16 (0.2%)	0 (0.0%)	16 (0.3%)		1 (0.1%)	15 (0.3%)	
**Clinical characteristics**							
Present with any symptoms	4,533 (68.9%)	178 (86.8%)	4,355 (68.3%)	<0.0001	1,484 (85.9%)	3,049 (62.8%)	<0.0001
Presenting symptoms							
Cough	3,072 (46.7%)	114 (55.6%)	2,958 (46.4%)	0.032	975 (56.5%)	2,097 (43.2%)	<0.0001

Fever	2,894 (44.0%)	102 (49.8%)	2,792 (43.8%)	0.227	1,017 (58.9%)	1,877 (38.7%)	<0.0001
Runny nose	2,174 (33.0%)	42 (20.5%)	2,132 (33.4%)	0.001	453 (26.2%)	1,721 (35.4%)	<0.0001
Malaise	1,671 (25.4%)	114 (55.6%)	1,557 (24.4%)	0.000	770 (44.6%)	901 (18.6%)	<0.0001
Headache	1,447 (22.0%)	52 (25.4%)	1,395 (21.9%)	0.472	471 (27.3%)	976 (20.1%)	<0.0001
Sore throat	1,072 (16.3%)	35 (17.1%)	170 (82.9%)	0.909	286 (16.6%)	786 (16.2%)	0.551
Myalgia	1,030 (15.7%)	48 (23.4%)	982 (15.4%)	0.008	357 (20.7%)	673 (13.9%)	<0.0001
Anosmia	942 (14.3%)	12 (5.9%)	930 (14.6%)	<0.0001	202 (11.7%)	740 (15.2%)	<0.0001
Shortness of breath	858 (13.0%)	121 (59.0%)	737 (11.7%)	<0.0001	556 (32.2%)	302 (6.2%)	<0.0001
Nausea/vomiting	755 (11.5%)	53 (25.9%)	702 (11.0%)	<0.0001	410 (23.7%)	345 (7.1%)	<0.0001
Ageusia	321 (4.9%)	10 (4.9%)	311 (4.9%)	0.006	72 (4.2%)	249 (5.1%)	<0.0001
Abdominal pain	343 (5.2%)	29 (14.2%)	314 (4.9%)	<0.0001	189 (10.9%)	154 (3.2%)	<0.0001
Diarrhoea	253 (3.8%)	11 (5.4%)	242 (3.8%)	0.491	104 (6.0%)	149 (3.1%)	<0.0001
Number of symptoms, median (IQR)	2 (0-4)	3 (1-5)	2 (0-4)	<0.0001	3 (1-5)	1 (0-4)	<0.0001
Clinical diagnosis with pneumonia	319 (4.9%)	68 (33.2%)	251 (3.9%)	<0.0001	279 (16.2%)	40 (0.8%)	<0.0001
Number of comorbidities				<0.0001			<0.0001
0	5,638 (85.6%)	81 (39.5%)	5,557 (87.1%)		1,124 (65.1%)	4,514 (93.9%)	
1	707 (10.7%)	70 (34.2%)	637 (10.0%)		432 (25.0%)	275 (5.7%)	
>1	238 (3.6%)	54 (26.3%)	184 (2.9%)		171 (9.9%)	67 (1.4%)	
Type of comorbidity							
Hypertension	539 (8.2%)	68 (33.2%)	471 (7.4%)	<0.0001	332 (19.2%)	207 (4.3%)	<0.0001
Diabetes	365 (5.5%)	59 (28.8%)	306 (4.8%)	<0.0001	247 (14.3%)	118 (2.4%)	<0.0001
Cardiac diseases	187 (2.8%)	31 (15.1%)	156 (2.5%)	<0.0001	122 (7.1%)	65 (1.3%)	<0.0001
COPD	48 (0.7%)	11 (5.4%)	37 (0.6%)	<0.0001	41 (2.4%)	7 (0.1%)	<0.0001
Chronic kidney diseases	46 (0.7%)	16 (7.8%)	30 (0.5%)	<0.0001	36 (2.1%)	10 (0.2%)	<0.0001
Malignancy	20 (0.3%)	4 (2.0%)	16 (0.3%)	<0.0001	11 (0.6%)	9 (0.2%)	0.003
Immunocompromised	17 (0.3%)	0 (0.0%)	17 (0.3%)	0.459	11 (0.6%)	6 (0.1%)	<0.0001
Liver diseases	14 (0.2%)	4 (2.0%)	10 (0.2%)	<0.0001	11 (0.6%)	3 (0.1%)	<0.0001

*5,104 (78%) were PCR-confirmed, and 1,479 (22%) were Ag RDT-confirmed.
** Statistics were generated based on province-level data.
IQR: inter quartile range.
·· the Mann-Whitney U test was not performed due to identical median and IQR between group.

The mortality and hospitalisation rate increased with age (Table 2). Whilst age-specific mortality rate ranged from 1% (2/340) for 0-4 years to 16% (37/232) for ≥70 years, the age-specific hospitalisation rate ranged from 17% (37/215) for 0-4 years to 57% (131/232) for ≥70 years. Based on province, the mortality and hospitalisation rate ranged from 2% (13/658) and 13% (88/658) in East Nusa Tenggara to 4% (93/2,146) and 36% (775/2,146) in Lampung. Details on age-specific mortality and hospitalisation rate can be seen in Table 2.

Table 2: Province and age-specific mortality and hospitalisation rate with confidence limits among COVID-19 confirmed cases in five rural provinces in Indonesia, 1 January to 31 July 2021

	N total	Mortality		Hospitalisation	
		N	% (95% CI)	N	% (95% CI)
Province					
Lampung	2,146	93	4.33 (3.55-5.28)	775	36.1 (34.1-38.2)
Gorontalo	1,356	34	2.51 (1.80-3.49)	340	25.1 (22.8-27.5)
Central Sulawesi	987	27	2.74 (1.88-3.96)	258	26.1 (23.5-29.0)
Southeast Sulawesi	1,436	38	2.65 (1.93-3.62)	266	18.5 (16.6-20.6)
East Nusa Tenggara	658	13	1.98 (1.15-3.38)	88	13.4 (11.0-16.2)
Age group (years)					
0-4	215	2	0.93 (0.23-3.64)	37	17.2 (12.7-22.9)
5-9	112	0	NA	14	12.5 (7.5-20.0)
10-19	498	1	0.20 (0.03-1.41)	65	13.1 (10.4-16.3)
20-29	1,385	11	0.79 (0.44-1.43)	213	15.4 (13.6-17.4)
30-39	1,382	12	0.87 (0.49-1.52)	249	18.0 (16.1-20.1)
40-49	1,095	23	2.10 (1.40-3.14)	273	24.9 (22.5-27.6)
50-59	1,064	57	5.36 (4.15-6.88)	446	41.9 (39.0-44.9)
60-69	576	62	10.76 (8.48-13.57)	297	51.6 (47.5-55.6)
≥70	232	37	15.95 (11.78-21.24)	131	56.5 (50.0-62.7)

NA: Not Applicable due to 0 value of observation among deceased cases aged 5-9 years old.

Factors associated with COVID-19-related mortality and hospitalisation

In bivariable mixed effects logistic regression analysis (Supplementary Table 3), the risk of death was associated with older age, clinical diagnosis with pneumonia, number of pre-existing comorbidities, hypertension, diabetes, cardiac disease, COPD, chronic kidney disease, liver diseases, malignancy, lower number of nurses per 100,000 population, and higher number of midwives per 100,000 population. The risk of being hospitalised was associated with older age, clinical diagnosis with pneumonia, number of pre-existing comorbidities, hypertension, diabetes, cardiac disease, COPD, chronic kidney disease, liver diseases, malignancy, immunocompromised, and lower number of nurses per 100,000 populations.

Table 3: Mixed effects logistic regression multivariable models showing factors associated with risk of COVID-19 mortality and hospitalisation in five rural provinces in Indonesia, 1 January to 31 July 2021

	Mortality		Hospitalisation	
	**aOR (95% CI)**	**p value**	**aOR (95% CI)**	**p value**
**Demographics**				
Age group, years				
0-19	1 (reference)		1 (reference)	
20-29	1.5 (0.4-5.8)	0.537	1.1 (0.9-1.5)	0.354
30-39	1.9 (0.5-6.8)	0.342	1·24 (1.0-1.6)	0.119
40-49	3.1 (0.9-10.6)	0.076	**1.7 (1.3-2.2)**	**<0.0001**
50-59	**6.5 (2.0-21.3)**	**0.002**	**2.9 (2.2-3.8)**	**<0.0001**
60-69	**10.8 (3.2-35.8)**	**<0.0001**	**3.3 (2.4-4.4)**	**<0.0001**
≥70	**18.3 (5.3-63.2)**	**<0.0001**	**3.7 (2.5-5.5)**	**<0.0001**
Sex				
Female	1 (reference)		1 (reference)	
Male	1·3 (0.9-1.8)	0.193	0.9 (0.8-1.0)	0.057
**Clinical characteristics**				
Clinical diagnosis with pneumonia	**8.0 (5.3-12.0)**	**<0.0001**	**15.3 (10.7-21.8)**	**<0.0001**
Type of comorbidity				
Hypertension	1.4 (0.9-2.1)	0.123	**2.4 (1.9-3.0)**	**<0.0001**
Diabetes	**2.0 (1.3-3.2)**	**0.002**	**2.2 (1.7-2.9)**	**<0.0001**
Cardiac diseases	1.6 (0.9-2.9)	0.119	**2.1 (1.4-3.1)**	**<0.0001**
COPD	1.9 (0.7-4.8)	0.184	**7.5 (2.3-24.1)**	**0.001**
Chronic kidney diseases	**6.1 (2.5-14.6)**	**<0.0001**	**3.6 (1.6-8.1)**	**0.002**
Liver diseases	**10.3 (1.8-58.7)**	**0.008**	**7.5 (1.8-31.0)**	**0.006**
Malignancy	**7.4 (1.5-36.9)**	**0.015**	**3.4 (1.1-10.4)**	**0.029**
Immunocompromised	··	··	**6.9 (2.4-20.2)**	**<0.0001**
**Health care workers**				
Number of doctors per 100,000 population	··	··	··	··
Number of nurses per 100,000 population	1.2 (0.9-1.6)	0.185	0.8 (0.6-1.1)	0.139
Number of midwives per 100,000 population	1.5 (0.6-3.8)	0.382	··	··
Number of public health officers per 100,000 population	1.0 (0.8-1.3)	0.698	··	··

Province was treated as the random effect variable.
aOR: adjusted odds ratio.
·· the variable did not enter the multivariable model therefore statistics were not estimated.

Based on the first multivariable mixed effects logistic regression model (Table 3), the risk of death increased for age groups 50-59 years (aOR 6.5, 95%CI 2.0-21.3), 60-69 years (aOR 10.8, 95%CI 3.2-35.8), and ≥70 years (aOR 18.3, 95%CI 5.3-63.2), compared to <20 years; for cases with pre-existing diabetes (aOR 2.0, 95%CI 1.3-3.2), chronic kidney disease (aOR 6.1, 95%CI 2.5-14.6), liver diseases (aOR 10.3, 95% CI 1.8-58.9), or malignancy (aOR 7.4,

95% CI 1.5-36.9); for patients who had pneumonia (aOR 8.0, 95%CI 5.3-12.0), compared to those without pre-existing comorbidities.

The risk of being hospitalised increased for age groups 40-49 years (aOR 1.7, 95%CI 1.3-2.2), 50-59 years (aOR 2.9, 95%CI 2.2-3.8), 60-69 years (aOR 3.3, 95%CI 2.4-4.4), and ≥70 years (aOR 3.7, 95%CI 2.5-5.5), compared to <20 years; for cases with pre-existing hypertension (aOR 2.4, 95%CI 1.9-3.0), diabetes (aOR 2.2, 95%CI 1.7-2.9), cardiac disease (aOR 2.1, 95% CI 1.4-3.1), COPD (aOR 7.5, 95% CI 2.3-24.0), chronic kidney disease (aOR 3.6, 95%CI 1.6-8.2), liver diseases (aOR 7.5, 95% CI 1.8-31.0), malignancy (aOR 3.5, 95% CI 1.1-10.5), or immunocompromised (aOR 6.9, 95% CI 2.4-20.2); for patients who had pneumonia (aOR 15.3, 95%CI 10.7-21.8).

In the second multivariable model (Supplementary Table 4), we found that both the risk of death and hospitalisation increased with the number of pre-existing comorbidities, after controlling for age, sex, pneumonia, and number of health care workers. Sex and number of health care workers at province-level were not associated with COVID-19-related hospitalisation and mortality. Sex was not found to be an effect modifier in either model.

Discussion

This retrospective cohort study described the clinical characteristics and factors associated with risk of COVID-19-related mortality and hospitalisation based on epidemiological surveillance data of confirmed COVID-19 cases reported from five rural provinces of Indonesia, during January to July 2021. This analysis was one of the first COVID-19 epidemiological studies reporting data from rural settings of Indonesia. The observed disease pattern broadly reflects that reported globally, with patients usually presenting multiple symptoms of fever, cough, malaise and/or shortness of breath. The overall mortality and hospitalisation rate was 3% (205/6,583) and 26% (1,727/6,583), respectively. The age-specific mortality and hospitalisation rate for children under five years old was 0.9% (2/340) and 17% (37/215), respectively. The mortality and hospitalisation rate were highly heterogeneous, with the highest rate was reported in Lampung (4% mortality and 36% hospitalisation), and the lowest rate in East Nusa Tenggara (2% mortality and 13% hospitalisation). Higher risk of mortality and hospital admission was associated with increased age, clinical diagnosis with pneumonia, presence of pre-existing diabetes, chronic kidney disease, liver disease, and malignancy. Presence of pre-existing hypertension, cardiac diseases, COPD, and immunocompromised conditions were associated with higher risk of hospitalisation, but not associated with mortality. Gender and number of health care workers at province-level were not associated with COVID-19-related mortality and hospitalisation.

The overall mortality rate in these five rural provinces was higher than the mortality rate reported from Jakarta (3% vs 1.5%), potentially due to a higher prevalence of at least one comorbidity in these settings (14% vs 0.7%). However, our findings suggest that the hospitalisation rate was slightly lower than in Jakarta (26% vs 33%), possibly due to a lower level of transmission, a lower health care capacity, and a lower access to health care facility in these five provinces. For comparison, as presented in Supplementary Table 1, the highest capacity in these settings was seen in Southeast Sulawesi, with 13 doctors per 100,000 population, 37 nurses per 100,000 population, and 13 midwives per 100,000 population. By contrast, Jakarta has 45 doctors per 100,000 population, 48 nurses per 100,000 population, and 31 midwives per 100,000 population. In addition, as previously reported in Jakarta [15], the inequality of COVID-19 vaccine coverage may also contribute to the mortality and hospitalisation rate in Jakarta, but this was not evaluated in the present study. Further study assessing association between health care capacity, including vaccine coverage, with COVID-19-related mortality and hospitalisation is needed to better understand Indonesia's health system resilience in responding the current and future global health crisis.

Consistent with a recent finding from an urban area of Indonesia, Jakarta [15], the mortality rate for children under five years old in these rural settings was very low (<1%). This finding was also consistent with several other studies from China [25], Brazil [29,30], Uganda [16], and South Africa [17] that have reported COVID-19-related deaths among children under 5 years to be rare. Moreover, a review of data from US, Korea and Europe in the early phase of epidemic estimated the overall case fatality rate among children (0–19 years old) to be as low as 0.03 per 100 000 (44/42 846) [31].

Our findings suggest that COVID-19-related mortality and hospitalisation rates varied across the five provinces. For example, compared to in East Nusa Tenggara, the rate in Lampung was 2 times (4% vs 2%) for mortality and 2.8 times (36% vs 13%) for hospitalisation. The observed different risks of COVID-19-related mortality and hospitalisation could be due to several factors such as different prevalence of older and comorbid population, as well as different level of health care capacity across the provinces. Although our study found an absence of association between number of health care workers at province-level with COVID-19-related mortality and hospitalisation, a previous nationwide ecological study in Indonesia, suggested that COVID-19 has disproportionately affected district with high proportion of elder population, high prevalence of diabetes mellitus, lower doctor to population ratio, higher life expectancy at birth, and lower level of formal education [32]. Other health care factors such as quality of COVID-19 diagnosis and case management may also affect the COVID-19 heterogeneity, but these factors were not evaluated in the current study.

Risk of mortality and hospitalisation increased with the presence of one or more comorbidities, especially pre-existing diabetes, chronic kidney diseases, liver diseases, or malignancy, and clinical pneumonia. The mortality risk increased by 102%, 505%, 934%, 637%, and 700% for people with pre-existing diabetes, chronic kidney diseases, liver diseases, malignancy, and a clinical diagnosis with pneumonia, respectively. The presence of pre-existing hypertension, cardiac diseases, COPD, or immunocompromised conditions also increased risk of hospitalisation by 136%, 108%, 646%, and 593%, respectively, but was not associated with increased risk of death. In addition, mortality and hospitalisation risk were increased for people which were clinically diagnosed with pneumonia. These findings are consistent with evidence from previous studies suggesting that pre-existing comorbidities, including cardiovascular or cerebrovascular diseases, hypertension, and diabetes mellitus, were associated with poorer COVID-19 outcomes [5–7, 33]. Although chronic comorbidities among COVID-19 cases in these five rural provinces (14%) were less frequent than reported in more developed settings including North America [5,6], South America [23], and Europe [7], this finding could reflect the under-reporting of comorbidities by patients, or under-diagnosis due to variable access to quality health services. Finally, obesity has also been recognised as an important risk factor of mortality [7,18], but we could not assess this due to absence of data for this variable.

This study has limitations. The retrospective design and reliance on routine surveillance data meant that data were incomplete or unavailable for some key baseline variables (e.g., date of symptom onset, date of hospital admission and outcome, vital signs, and disease severity classification). The imperfect contact tracing, testing, and reporting activities could result in underreporting of cases, especially for asymptomatic and mild cases which thus results in overestimation of rates of mortality and hospitalisation rate. Comorbidities were often self-reported or could be under-diagnosed, potentially resulting in underreporting and hence underestimation of effect sizes. Findings from this study may not reflect the mortality and hospitalisation rate, and risk factors associated with COVID-19-related outcomes in other LMIC settings with different demographics, non-infectious diseases prevalence, and different health care capacity.

In conclusion, clinical characteristics and risk factors associated with severe COVID-19 outcomes in five rural provinces in Indonesia are broadly similar to those in the urban province of Jakarta, and in more developed countries in North America, Europe, and Asia, dominated by advanced age and comorbidities. Heterogeneity of mortality and hospitalisation rate in the studied areas was likely driven by the different age structure and prevalence of pre-existing comorbidities. The findings highlight the need for enhanced context-specific public health action to reduce mortality and hospitalisation risk among older and comorbid rural populations,

and improved care and treatment for the cases. Although health care worker capacity was not associated with mortality and hospitalisation in this study, future nationwide study assessing association between health care capacity and COVID-19 impacts is needed to better guide improvement of health systems resilience in the future.

Author's contributions

HS was the principal investigator of this study. HS designed the study, did the analysis, and had full access to all of the data in the study and take responsibility for the integrity of the data and the accuracy of the data analysis. AME, MR, WN, JADH contributed to data collection and verification. ENM, YYS, SS, RR, IM, and HW supervised the data collection and verification. CYP, WAH, and HW contributed to funding acquisition for the SISUGI development and implementation. HS, LF and DR obtained the ethical approval. HS, CYP, IRFE, and WAH drafted the paper. All authors critically revised the manuscript for important intellectual content and all authors gave final approval for the version to be published.

Acknowledgments

We acknowledge South Asia Field Epidemiology and Technology Network (SAFETYNET) for their support in the development and implementation of SISUGI, and all health care workers involved in the care for the COVID-19 patients, as well as those involved in the field data collection.

References

1. World Health Organization. WHO Coronavirus Disease (COVID-19) Dashboard. Available: https://covid19.who.int [Accessed 22 June 2022].
2. Dong E, Du H, Gardner L. An interactive web-based dashboard to track COVID-19 in real time. Lancet Infect Dis. 2020;20(5):533–4. Available from: http://dx.doi.org/10.1016/S1473-3099(20)30120-1
3. Petrilli CM, Jones SA, Yang J, Rajagopalan H, et al. Factors associated with hospital admission and critical illness among 5279 people with coronavirus disease 2019 in New York City: Prospective cohort study. BMJ. 2020;369.
4. Lewnard JA, Liu VX, Jackson ML, Schmidt MA, et al. Incidence, clinical outcomes, and transmission dynamics of severe coronavirus disease 2019 in California and Washington: Prospective cohort study. BMJ. 2020;369(9).
5. Richardson S, Hirsch JS, Narasimhan M, Crawford JM, et al. Presenting Characteristics, Comorbidities, and Outcomes among 5700 Patients Hospitalized with COVID-19 in the New York City Area. JAMA - J Am Med Assoc. 2020;323(20):2052–9.
6. Harrison SL, Fazio-Eynullayeva E, Lane DA, Underhill P, Lip GYH. Comorbidities associated with mortality in 31,461 adults with COVID-19 in the United States: A federated electronic medical record analysis. PLoS Med 2020;17(9):1–11.
7. Docherty AB, Harrison EM, Green CA, Hardwick HE, et al. Features of 20 133 UK patients in hospital with covid-19 using the ISARIC WHO Clinical Characterisation Protocol: Prospective observational cohort study. BMJ. 2020;369(March):1–12.

8. Zhou F, Yu T, Du R, Fan G, et al. Clinical course and risk factors for mortality of adult inpatients with COVID-19 in Wuhan, China: a retrospective cohort study. Lancet 2020;395(10229):1054–62.
9. Surendra H, IRF E, Djaafara BA, Ekawati LL, et al. Clinical characteristics and mortality associated with COVID-19 in Jakarta , Indonesia : A hospital-based retrospective cohort study. Lancet Reg Heal - West Pacific. 2021;9:100108.
10. Oliveira EA, Colosimo EA, Simões e Silva AC, Mak RH, Martelli DB, Silva LR, et al. Clinical characteristics and risk factors for death among hospitalised children and adolescents with COVID-19 in Brazil: an analysis of a nationwide database. Lancet Child Adolesc Heal. 2021;5(8):559–68.
11. Karmakar M, Lantz PM, Tipirneni R. Association of Social and Demographic Factors with COVID-19 Incidence and Death Rates in the US. JAMA Netw Open. 2021;4(1):1–12.
12. Chen JT, Krieger N. Revealing the unequal burden of COVID-19 by income, race/ethnicity, and household crowding: US county versus zip code analyses. J Public Heal Manag Pract. 2021;27(1):S46–56.
13. Mena GE, Martinez PP, Mahmud AS, Marquet PA, et al. Socioeconomic status determines COVID-19 incidence and related mortality in Santiago, Chile. Science (80-). 2021;372(6545).
14. Sousa BLA, Brentani A, Costa Ribeiro CC, Dolhnikoff M, et al. Non-communicable diseases, sociodemographic vulnerability and the risk of mortality in hospitalised children and adolescents with COVID-19 in Brazil: a cross-sectional observational study. BMJ Open. 2021;11(9):e050724.
15. Surendra H, Salama N, Lestari K, Adrian V, et al. Pandemic inequity in a megacity: a multilevel analysis of individual, community, and health care vulnerability risks for COVID-19 mortality in Jakarta, Indonesia. BMJ Global Health 2022;7:e008329.
16. Kirenga B, Muttamba W, Kayongo A, Nsereko C, et al. Characteristics and outcomes of admitted patients infected with SARS-CoV-2 in Uganda. BMJ Open Respir Res. 2020;7(1).
17. Boulle AA, Davies M, Hussey H, Morden E, et al. Risk factors for COVID-19 death in a population cohort study from the Western Cape Province , South Africa. Clin Infect Dis. 2020;1–31.
18. Nachega J.B, Ishoso D.K, Otokoye J.O, Hermans M.P, et al. Clinical characteristics and outcomes of patients hospitalized for COVID-19 in Africa: early insights from the democratic republic of the Congo. Am J Trop Med Hyg. 2020; 3-5
19. https://covid19.go.id/peta-sebaran. [Accessed 17 November 2022].
20. Sistem Informasi Surveilans Epidemiologi. Available from: https://sisugi.com/
21. Keputusan Menteri Kesehatan Republik Indonesia Nomor HK.01.07/MENKES/446/2021. Available from: https://covid19.go.id/artikel/2021/02/09/keputusan-menteri-kesehatan-republik-indonesia-nomor-hk0107menkes4462021
22. Badan Pusat Statistik. Peraturan Badan Pusat Statistik Nomor 120 Tahun 2020 tentang Klasifikasi Desa Perkotaan dan Perdesaan di Indonesia 2020 Buku 1 Sumatera. Jakarta 2021. Available from: https://www.bps.go.id/publication/2021/05/26/eb5af575283fd897b51a487d/peraturan-badan-pusat-statistik-nomor-120-tahun-2020-tentang-klasifikasi-desa-perkotaan-dan-perdesaan-di-indonesia-2020-buku-1-sumatera.html
23. Badan Pusat Statistik. Peraturan Badan Pusat Statistik Nomor 120 Tahun 2020 tentang Klasifikasi Desa Perkotaan dan Perdesaan di Indonesia 2020 Buku 3 Bali, Nusa Tenggara, Kalimantan, Sulawesi, Maluku dan Papua. Jakarta 2021. https://www.bps.go.id/publication/2021/05/26/7eba842e1090fccf23f34e71/peraturan-kepala-badan-pusat-statistik-nomor-120-tahun-2020-tentang-klasifikasi-desa-perkotaan-dan-perdesaan-di-indonesia-2020-buku-3-bali-nusa-tenggara-kalimantan-sulawesi-maluku-dan-papua.html
24. WHO. Clinical management of COVID-19: interim guidance 27. WHO, Geneva May

2020: 2020.

25. Kementrian Kesehatan Republik Indonesia. Pedoman pencegahan dan pengendalian coronavirus disease (COVID-19). vol. 5. Kementrian Kesehatan Republik Indonesia, Jakarta 2020: 1-135. Available from: https://covid19.go.id/artikel/2020/07/28/pedoman-pencegahan-dan-pengendalian-coronavirus-disease-covid-19-revisi-ke-5
26. Badan Pusat Statistik. Jumlah Penduduk Hasil Sensus Penduduk 2020 menurut Wilayah dan Jenis Kelamin. Available from: https://www.bps.go.id/subject/12/kependudukan.html#subjekViewTab5.
27. von Elm E, Altman DG, Egger M, Pocock SJ, et al. The Strengthening the Reporting of Observational Studies in Epidemiology (STROBE) statement: guidelines for reporting observational studies. J Clin Epidemiol. 2008;61(4):344–9.
28. Lu X, Zhang L, Du H, Zhang J, et al. SARS-CoV-2 infection in children. N Engl J Med. 2020; 382: 1663-1665.
29. Baqui P, Bica I, Marra V, Ercole A, et al. Ethnic and regional variations in hospital mortality from COVID-19 in Brazil: a cross-sectional observational study. Lancet Glob Heal. 2020;8(8):e1018–26.
30. Santos MM, Lucena EES, Lima KC, Brito AAC, et al. Survival and predictors of deaths of patients hospitalised due to COVID-19 from a retrospective and multicentre cohort study in Brazil. Epidemiol Infect. 2020;148:e198.
31. Bhopal S, Bagaria J, Bhopal R. Children's mortality from COVID-19 compared with all-deaths and other relevant causes of death: epidemiological information for decision-making by parents, teachers, clinicians and policymakers Governments. Public Health. 2020; 185: 19-20
32. Surendra H, Paramita D, Arista NN, Putri AI, et al. Heterogeneity and district-level factors associated with COVID-19 mortality during three epidemic waves in Indonesia: a nationwide ecological study. MedRxiv 2022.
33. Guan W-j, Liang W-h, Zhao Y. et al. Comorbidity and its impact on 1590 patients with Covid-19 in China: a nationwide analysis. Eur Respir J. 2020; 552000547.

Figure 1: Map showing five study sites in East Nusa Tenggara, Gorontalo, Central Sulawesi, Southeast Sulawesi, and Lampung province, and spatial distribution of cumulative COVID-19 cases by district. COVID-19 aggregated data were obtained from https://data.covid19.go.id/.

Figure 2: Study flow chart and completeness of key variables.

Figure 3: Presenting symptoms (A) and comorbidity (B) among symptomatic cases in 5 provinces in Indonesia.

Figure 1

Click here to access/download;Figure;Figure 1 Study sites_1200_frame.tiff

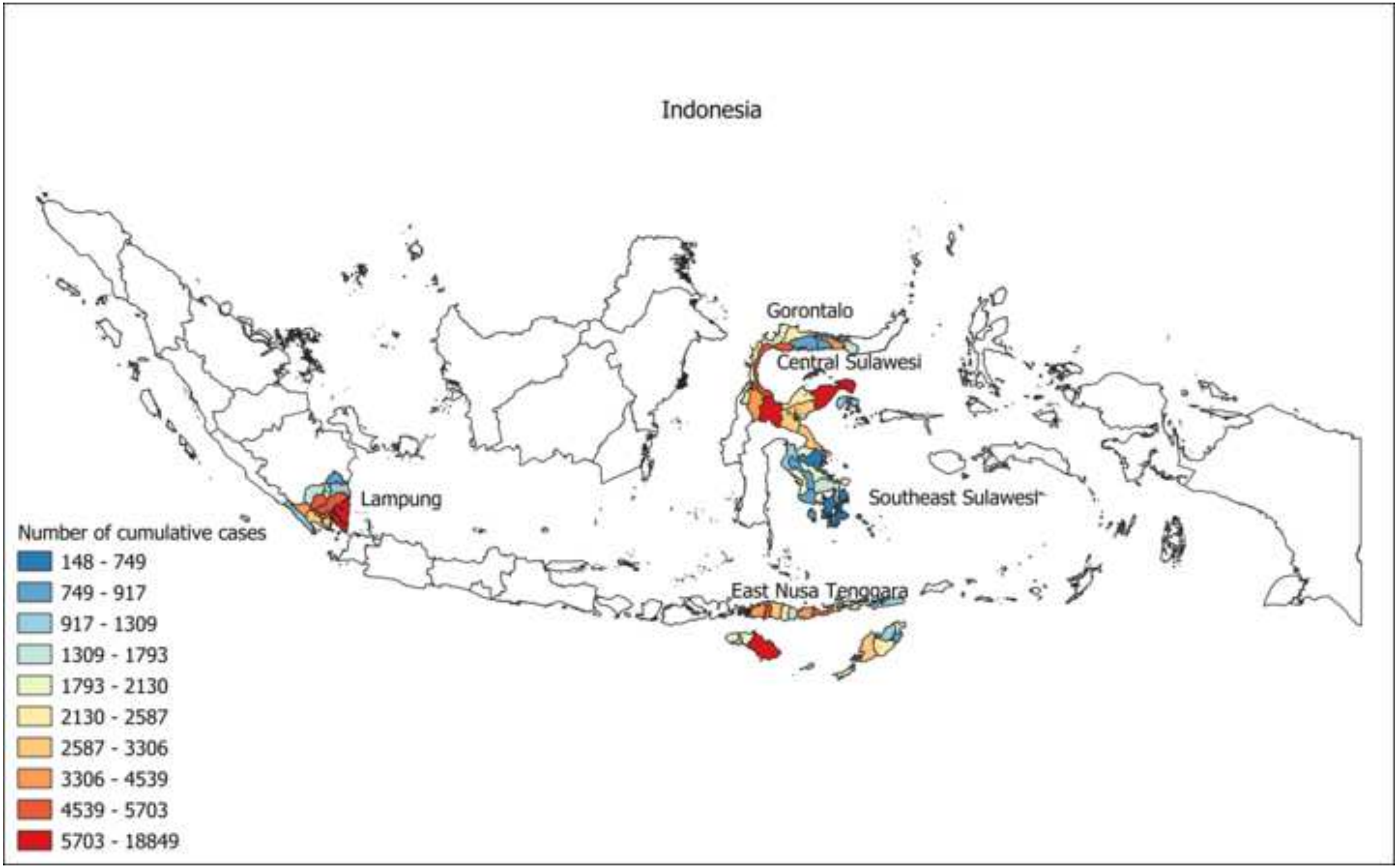

Figure 2

Click here to access/download;Figure;Figure 2_001.tif

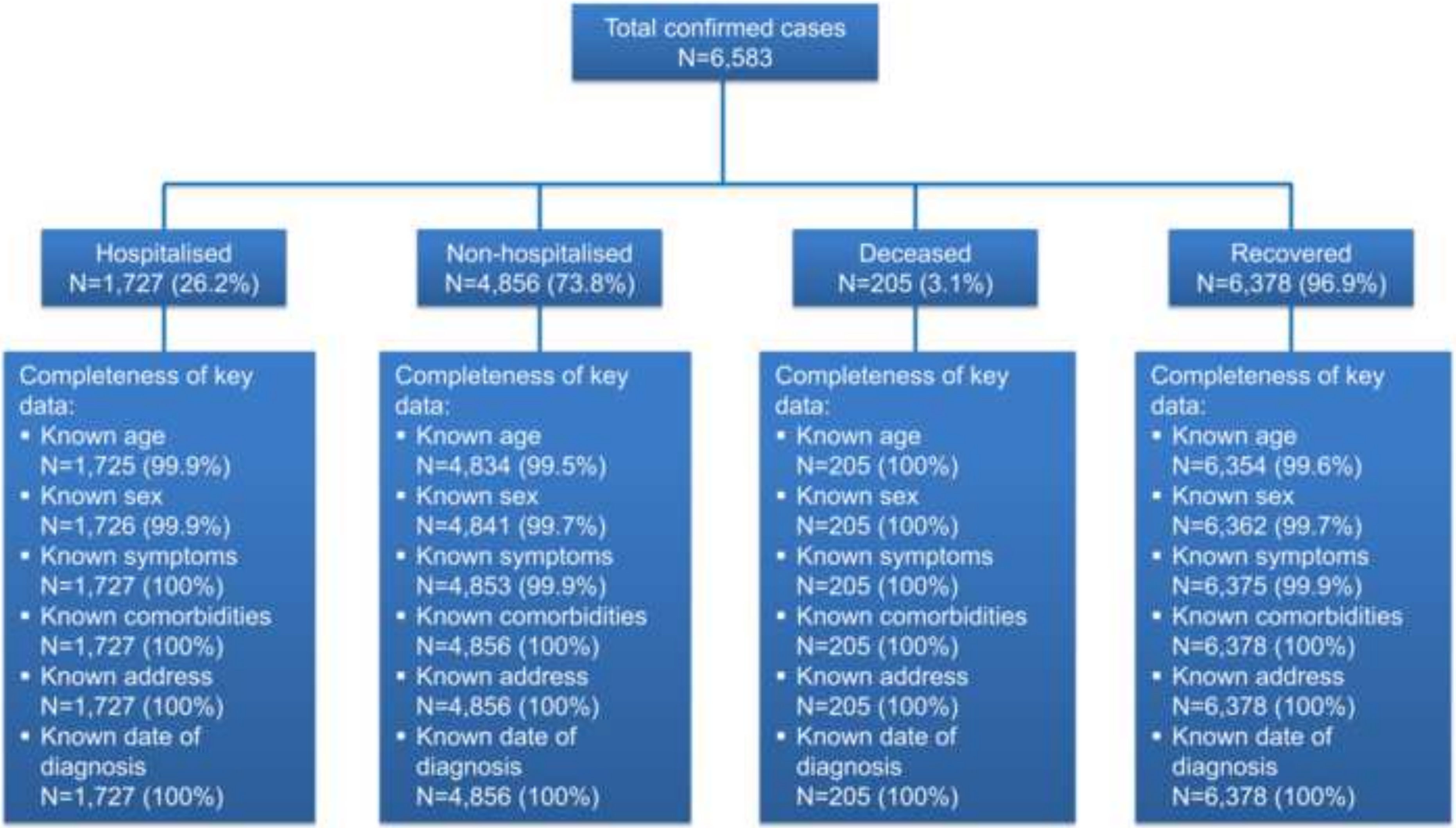

Figure 3

Click here to access/download;Figure;Figure 3_001.tif

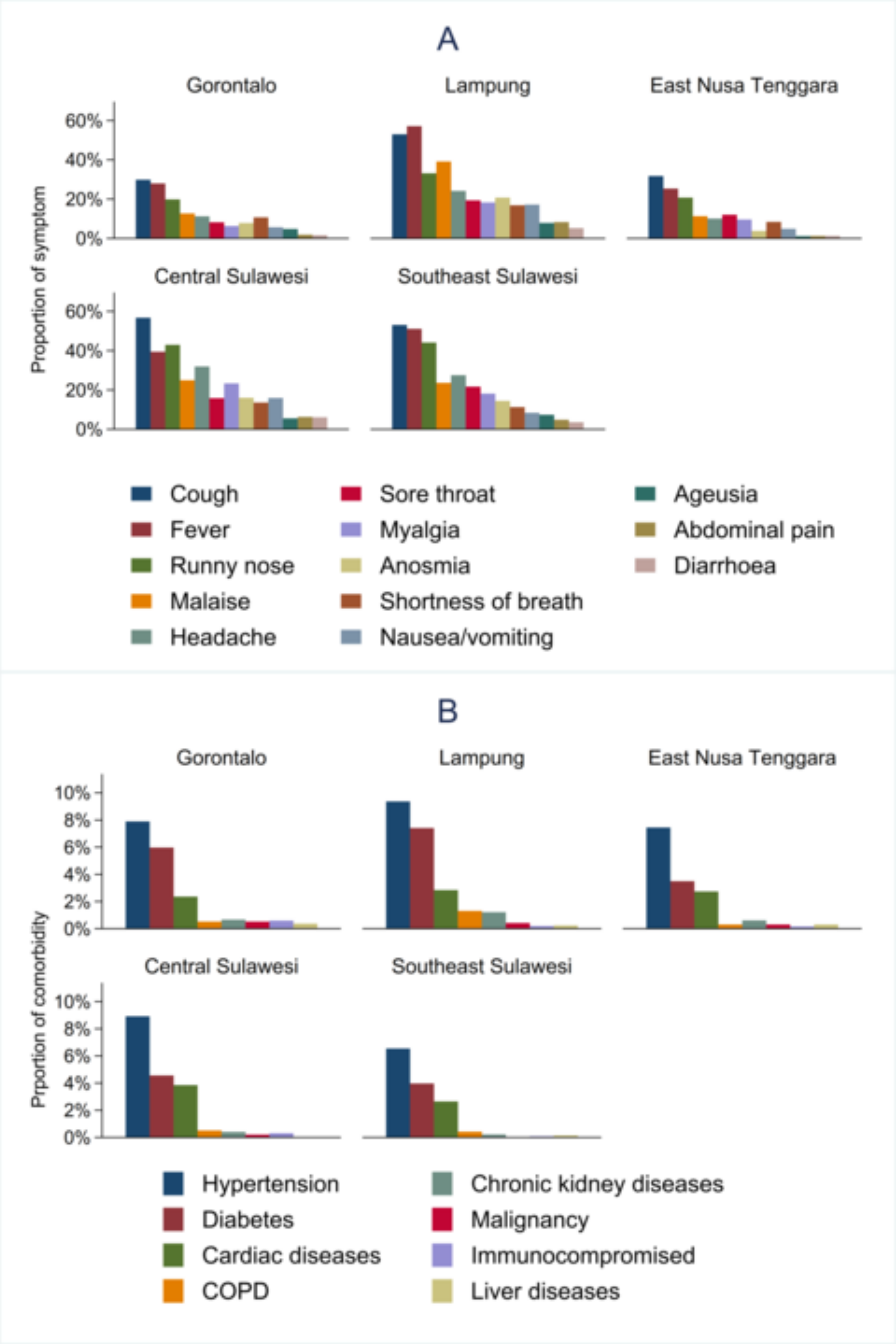

Click here to access/download
Supporting Information
Surendra et al. supplementary data_v1.0.docx
