# Supplementary material for: Clinical characteristics and factors associated with COVID-19-related mortality and hospital admission during the first two epidemic waves in 5 rural provinces in Indonesia: A retrospective cohort study
Source: PLoS One. 2023 Mar 30;18(3):e0283805. doi: 10.1371/journal.pone.0283805 (PMC10062642; doi:10.1371/journal.pone.0283805)
Supplement: Attachment — Submitted filename: Response to reviewer comments_R2.docx [file pone.0283805.s009.docx]

To Editor *PLOS ONE*

March 16^th^, 2023

Dear Editor,

We would like to thank the reviewers for their very positive and useful comments and have attached a revised manuscript of **“Clinical characteristics and factors associated with COVID-19-related mortality and hospital admission during the first two epidemic waves in 5 rural provinces in Indonesia: a retrospective cohort study”** (PONE-D-22-31666).

We would like to also specifically state that the data analysed in this study contain de-identified dataset own by the Indonesian Epidemiologist Association/Perhimpunan Ahli Epidemiologi Indonesia. After publication, the datasets used for this study will be made available to others on reasonable requests to the corresponding author and the Indonesian Epidemiologist Association (email: [info@paei.or.id](mailto:info@paei.or.id)), including a detailed research proposal, study objectives and statistical analysis plan.

Please find a point-by-point response to the reviewer comments appended below.

Thank you for your consideration.

Yours sincerely,

On behalf of co-authors,

Henry Surendra, PhD

Email: [henrysurendra.15@gmail.com](mailto:henrysurendra.15@gmail.com) or [henry.surendra@monash.edu](mailto:henry.surendra@monash.edu)

**Point by point response to reviewers’ comments**

**Editor comments:**

Journal requirements:

Please review your reference list to ensure that it is complete and correct. If you have cited papers that have been retracted, please include the rationale for doing so in the manuscript text, or remove these references and replace them with relevant current references. Any changes to the reference list should be mentioned in the rebuttal letter that accompanies your revised manuscript. If you need to cite a retracted article, indicate the article’s retracted status in the References list and also include a citation and full reference for the retraction notice.

**Author response:** We thank the editor for their very positive feedback. We have reviewed the accuracy and completeness of our reference list and confirm that there are no retracted papers cited in this manuscript. Please find the point-by-point response to reviewer comment below:

**Reviewers' comments:**

Reviewer's Responses to Questions

**Comments to the Author**

1. If the authors have adequately addressed your comments raised in a previous round of review and you feel that this manuscript is now acceptable for publication, you may indicate that here to bypass the “Comments to the Author” section, enter your conflict of interest statement in the “Confidential to Editor” section, and submit your "Accept" recommendation.

Reviewer #1: All comments have been addressed

Reviewer #2: (No Response)

2. Is the manuscript technically sound, and do the data support the conclusions?

The manuscript must describe a technically sound piece of scientific research with data that supports the conclusions. Experiments must have been conducted rigorously, with appropriate controls, replication, and sample sizes. The conclusions must be drawn appropriately based on the data presented.

Reviewer #1: Yes

Reviewer #2: Yes

3. Has the statistical analysis been performed appropriately and rigorously?

Reviewer #1: Yes

Reviewer #2: Yes

4. Have the authors made all data underlying the findings in their manuscript fully available?

The [PLOS Data policy](https://apc01.safelinks.protection.outlook.com/?url=http%3A%2F%2Fwww.plosone.org%2Fstatic%2Fpolicies.action%23sharing&data=05%7C01%7Chsurendra%40oucru.org%7Cd9f39d77136447c421cf08db2046cad6%7C22514748bbda4cb89795bfa3fbeec018%7C0%7C0%7C638139260655094249%7CUnknown%7CTWFpbGZsb3d8eyJWIjoiMC4wLjAwMDAiLCJQIjoiV2luMzIiLCJBTiI6Ik1haWwiLCJXVCI6Mn0%3D%7C3000%7C%7C%7C&sdata=QgqSGC3898%2B9JqUvektfcoEJPEaB8mYqf9ThwOrORNs%3D&reserved=0) requires authors to make all data underlying the findings described in their manuscript fully available without restriction, with rare exception (please refer to the Data Availability Statement in the manuscript PDF file). The data should be provided as part of the manuscript or its supporting information, or deposited to a public repository. For example, in addition to summary statistics, the data points behind means, medians and variance measures should be available. If there are restrictions on publicly sharing data—e.g. participant privacy or use of data from a third party—those must be specified.

Reviewer #1: No

Reviewer #2: No

**Author response:** As previously requested by editor, we have stated the following statemen in our cover letter:

*“We would like to also specifically state that the data analysed in this study contain de-identified dataset own by the Indonesian Epidemiologist Association/Perhimpunan Ahli Epidemiologi Indonesia. After publication, the datasets used for this study will be made available to others on reasonable requests to the corresponding author and the Indonesian Epidemiologist Association (email:* [*info@paei.or.id*](mailto:info@paei.or.id)*), including a detailed research proposal, study objectives and statistical analysis plan.”*

5. Is the manuscript presented in an intelligible fashion and written in standard English?

PLOS ONE does not copyedit accepted manuscripts, so the language in submitted articles must be clear, correct, and unambiguous. Any typographical or grammatical errors should be corrected at revision, so please note any specific errors here.

Reviewer #1: Yes

Reviewer #2: Yes

**Reviewer #1:**

Authors have addressed all my concerns. Some minor comments were left, but the manuscript does not need to be sent back to me.

**Reviewer comment #1**

Query #2: Regarding immune status from previous infection. Please clarify your statement in the manuscript.

**Author response:** We thank the reviewer for this comment. We have now added the following statement in Limitations, Discussion Line 383-389:

*“This study has limitations. The retrospective design and reliance on routine surveillance data meant that data were incomplete or unavailable for some key baseline variables (e.g.,* *date of symptom onset, date of hospital admission and outcome, vital signs, previous SARS-CoV-2 infection, and disease severity classification*). *As the national COVID-19 vaccination program has just started in the middle of January 2021 and in 14 selected high-priority provinces, excluding our study settings, we were not able to assess the vaccine effectiveness in the present study.”*

**Reviewer comment #2**

Additional comments:
1. The data were form “1 January to 31 July 2021”. For such data the title is too general. Kindly state which epidemic wave the data were from.

**Author response:** We thank the reviewer for this comment. We have now emphasised that the data were from the first two epidemic waves in Indonesia. We have stated this in the title to make it more precise and accurate, and have also added the following sentence to give more nuance of the first two epidemic waves occurred in Indonesia:

“*The first epidemic wave occurred from 2 March 2020 to 30 April 2021, and the more intense second wave dominated by Delta variant peaked in July 2021*” (Background, Line 98-99)

**Reviewer comment #3**

2. It is 2023 already, while your data were from 2021. Please highlight the importance of having these data.

**Author response:** We thank the reviewer for this comment. We have now added the following sentences to highlight the importance of having these data:

*“The study findings will inform decision on prioritising clinical and public health interventions to reduce risk of hospital admission and mortality associated with COVID-19 in other rural settings of Indonesia.”* (Background, Line 123-125)

**Reviewer #2:**

**Reviewer comment #1**

Thank you to the authors for their effort to revise the manuscript. From my point of view, the manuscript is getting better and clearer after revision. However, I found that two comments from my previous review were not sufficiently addressed.
1). I can not find the STROBE checklist in the attachments file as mentioned by the authors. Please clarify this issue.

**Author response:** We thank the reviewer for their positive comments. We can confirm that we have submitted the STROBE checklist in the previous round. However, it seems that the checklist is only for editor review and was not sent out to the reviewers.

**Reviewer comment #2**

2). Commonly, a p-value is written as “<0.001" instead of "<0.0001". (Table 1, Table 3, S3 Table & S4 Table).

**Author response:** We thank the reviewer for this comment and apologise for the errors. We have now revised it as “<0.001" accordingly.
